# The asymmetry of working memory training transfer: A systematic review and meta-analysis

**DOI:** 10.3758/s13423-026-02994-5

**Published:** 2026-09-08

**Authors:** Jiayi Fu, Yuxin Zhou, Mingliang Yuan, Ying Cai

**Affiliations:** 1 Department of Psychology and Behavioral Science, Zhejiang University, Hangzhou, 310007 China; 2 State Key Laboratory of Cognitive Neuroscience and Learning and IDG/McGovern Institute for Brain Research, Beijing Normal University, Beijing, 100875 China; 3 Department of Psychology, School of Humanities and Social Sciences, Anhui Agricultural University, Hefei, 230036 China; 4 Zhejiang Key Laboratory of Neurocognitive Development and Mental Health, Zhejiang University, Hangzhou, 310007 China

**Keywords:** Working memory training, Transfer asymmetry, Meta-analysis, Cognitive development

## Abstract

Working memory (WM) training is widely used to enhance cognitive performance; however, its transfer to untrained tasks remains controversial. Traditional theories emphasize task similarity as the primary determinant of training transfer, but they cannot fully explain emerging evidence of asymmetric transfer across tasks. Two directional transfer hypotheses are proposed here to explain this asymmetry: the resource-based transfer advantage hypothesis predicts stronger transfer from more to less cognitively demanding tasks, whereas the ability-based transfer advantage hypothesis predicts stronger transfer from tasks engaging broader task-general abilities to tasks engaging task-specific narrower abilities. The contrast between span and updating paradigms provides an informative framework for distinguishing these accounts, because updating tasks are generally more cognitively demanding, whereas span tasks involve broader abilities. Accordingly, we conducted a three-level meta-analysis of 55 studies (208 effect sizes; *N* = 3,492). The results showed that updating training transferred reliably to span tasks (*g* = 0.176,* p* < .001), whereas span training did not reliably transfer to updating tasks (*g* = 0.048, *p* = .453), supporting the resource-based account. This advantage of updating training also extended to non-WM outcomes and was more pronounced at lower training doses, in non-adult samples, and with verbal stimuli. Together, these findings extend WM transfer theory beyond task similarity by highlighting the importance of cognitive demand and offer guidance for WM training design.

**Supplementary Information:**

The online version contains supplementary material available at https://doi.org/10.3758/s13423-026-02994-5.

## Introduction

Working memory (WM) refers to the capacity to temporarily store and manipulate information and serves as the foundation for a wide range of higher-order cognitive processes (Baddeley, 1992). The limited transfer of WM training to untrained tasks has long constrained its applications (Morrison & Chein, 2011; Zhao et al., 2022). Since Thorndike’s common elements theory was established in the 1900 s, task similarity has dominated transfer frameworks for more than a century, centering on similarity as a primary determinant of training transfer likelihood (Waris et al., 2015; Woodworth & Thorndike, 1901). Although its rationale is supported by findings that near transfer between high-similarity tasks is more reliable than far transfer between low-similarity tasks, persistent critiques of these theories’ explanatory limitations have been reported by meta-analyses (Soveri et al., 2017a; You et al., 2021). More recently, WM training studies have reported directional or asymmetric transfer effects – cases in which training on Task A transfers to Task B, but training on Task B does not comparably transfer back to Task A – even when the two tasks are closely matched in similarity (Liu et al., 2023; Roque-Gutierrez & Ibbotson, 2023). However, isolated reports are often underpowered and provide limited mechanistic clarity. A comprehensive review and meta-analysis is therefore necessary to establish whether transfer asymmetry is reliable in WM training and to evaluate mechanistic interpretations beyond task similarity.

### Similarity-based accounts

Stemming from Thorndike’s common element theory (Woodworth & Thorndike, 1901), several similarity-based working memory training frameworks have been developed in recent decades. For instance, the neural plasticity theory posits that training improves shared neural substrates between trained and untrained tasks, emphasizing the plasticity of domain-general frontoparietal networks crucial for transfer effects. This view has sparked extensive WM training research (Klingberg, 2010, 2016). Moreover, process-specific theory argues that transfer occurs only when training targets cognitive processes that are shared across tasks, providing a critical perspective to explain null transfer outcomes and prompting mechanistic investigation in this field (Dahlin et al., 2008a; Taatgen, 2013). A common feature of these frameworks is their emphasis on the overlap between tasks – in neural substrates or cognitive processes – as a primary determinant of transfer.

Crucially, similarity-based accounts are typically nondirectional: when two tasks are matched in overlap, they provide no principled basis for predicting that A→B transfer should systematically exceed B→A transfer, unless additional assumptions are introduced (e.g., differential modifiability of shared components, differences in measurement sensitivity, or strategy-mediated changes). Because such auxiliary assumptions are rarely specified a priori, similarity-based accounts often accommodate asymmetric findings only post hoc and remain theoretically underspecified with respect to when and why directionality should emerge. Asymmetric transfer has been reported in individual WM training studies (Liu et al., 2023; Roque-Gutierrez & Ibbotson, 2023), and asymmetry-like patterns have been suggested across studies (Cai et al., 2022; Schwarb et al., 2016). It therefore remains unresolved whether these asymmetries reflect the genuine directional properties of WM transfer or instead just arise from study-specific factors, such as sampling variability and methodological heterogeneity. This motivates a meta-analytic approach that can both quantify directionality and test theoretically motivated assumptions under which directionality should be expected.

### Two directional hypotheses beyond similarity: Ability generality and resource demand

One possibility is that transfer asymmetry reflects differences in the cross-task generality of the abilities strengthened by training. For example, n-back training has been reported to transfer to syntactic processing more than syntactic training transfers back to WM outcomes (Roque-Gutierrez & Ibbotson, 2023). This pattern has been interpreted as consistent with the idea that WM training engages more broadly applicable control components, whereas syntactic training relies more heavily on domain-specific mechanisms. Building on this logic and aligning with hierarchical views of cognitive abilities (Carroll, 1993), we consider an ability-based transfer advantage hypothesis: training that strengthens cross-task general (broad) components should transfer more readily to tasks that depend on those components along with additional specialized operations than vice versa. Importantly, “broad” versus “narrow” is defined here by the breadth of applicability across tasks/outcomes (cross-task generality), not by the number or complexity of operations required by a task. Empirical evidence remains too limited and heterogeneous to evaluate this directional claim systematically, which motivates a meta-analytic test using a contrast where broad/narrow can be operationalized transparently.

Another possibility is that transfer directionality reflects nested resource requirements: training on a higher-demand task may recruit a superset of cognitive/neural resources that is sufficient for performance on a lower-demand task, whereas the reverse does not hold true. In terms of motivating analogies, motor-learning studies report stronger transfer from dominant- to nondominant-limb training (Teixeira, 2000), which is often attributed to high bilateral recruitment during dominant-limb practice. Perceptual learning studies similarly report transfer from configuration to element learning but not the opposite; this is consistent with the proposal that configuration processing involves both elemental representations and higher-order integrative operations (Gong et al., 2022). These cross-domain findings are not treated as direct evidence for WM mechanisms; rather, they motivate a resource-based transfer advantage hypothesis for WM training that must be tested within WM paradigms where demand can be compared on a common footing.

### An informative test case within the working memory (WM) domain: Span versus updating

The ability-based account concerns the cross-task generality of trained components, whereas the resource-based account concerns the level and source of cognitive demand during task performance. Although conceptually distinct, these dimensions are often difficult to orthogonalize because training–transfer contrasts can shift both demand and generality simultaneously, with no fixed relationship across task designs. This creates an identification problem for interpreting directionality and may contribute to heterogeneous findings across individual studies – motivating meta-analytic synthesis rather than reliance on any single contrast. This challenge is further compounded in cross-domain comparisons, where neither “generality” nor “demand” has a common scale; for example, asymmetric transfer has been reported between inhibition and WM training (Liu et al., 2023), but such results are difficult to attribute uniquely to either account because the two dimensions are not directly comparable across domains. Accordingly, an especially informative test case is one in which ability generality and task demand can be defined transparently and placed in direct competition within a shared domain.

**Fig. 1:**
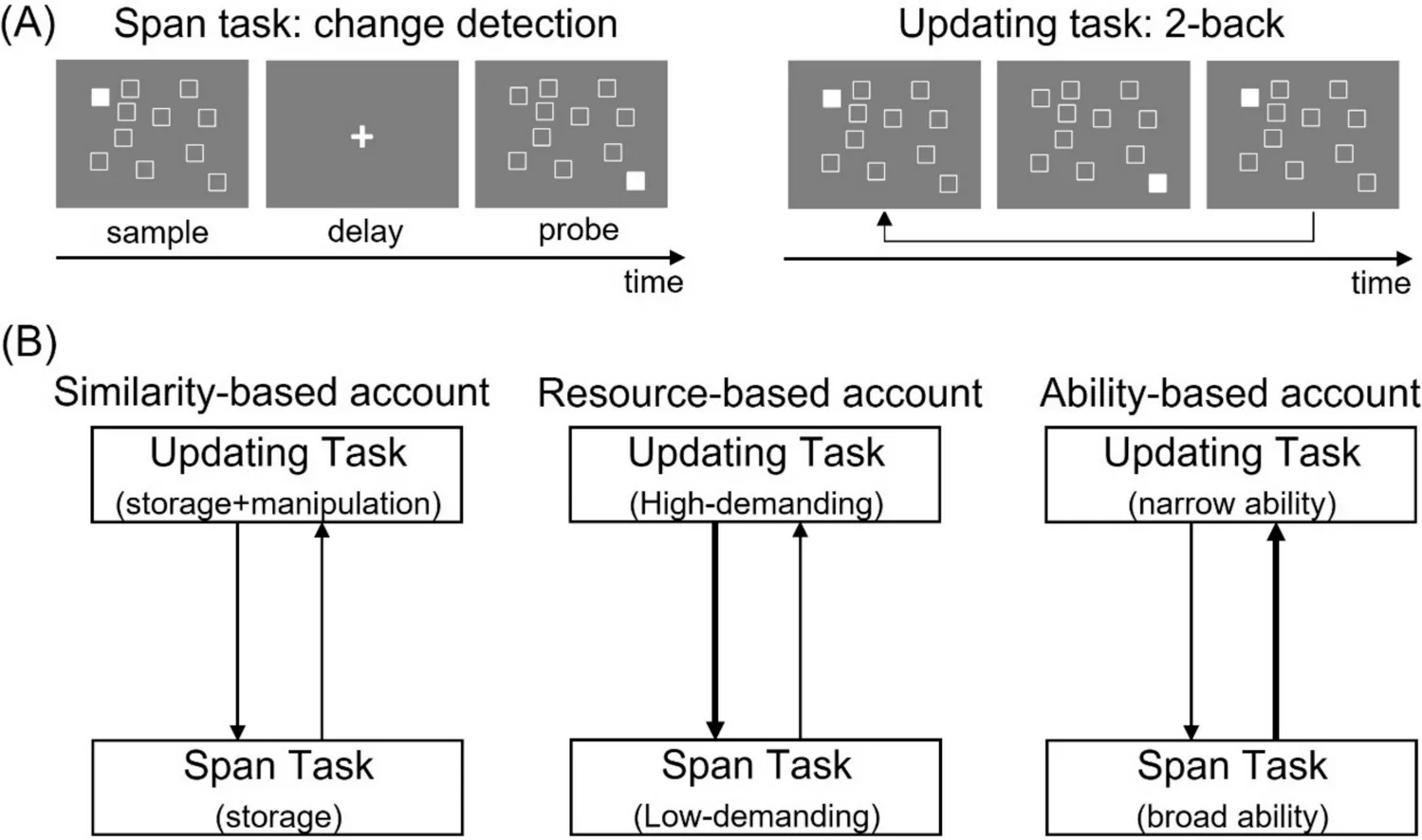
(**A**) Typical experimental procedure in span and updating tasks. (**B**) Three hypotheses for working memory training transfer directionality. Arrows denote transfer probabilities between tasks, with the bold lines indicating higher transfer probabilities

Two widely used WM paradigms – span and updating – provide such a test case. Span tasks require participants to recall or recognize memorized items after a short delay (Colom et al., 2006; Conway et al., 2005; Fig. 1A, left panel), whereas updating tasks require continuous monitoring and manipulation of dynamically maintained information (Cohen et al., 1997; Schwarb et al., 2016; Fig. 1A, right panel). Latent-variable and theoretical evidence suggests that span performance primarily reflects storage/maintenance, whereas updating reflects maintenance plus additional manipulation/control components (Aben et al., 2012; Burgoyne et al., 2025). Neuroimaging evidence likewise shows that both recruit frontoparietal systems, with updating eliciting stronger and more widespread engagement, which is consistent with greater cognitive demand (Rodríguez-Nieto et al., 2022; Wager & Smith, 2003).

Within our ability-based operationalization, maintenance is treated as a cross-task general core component required by both paradigms, whereas updating is treated as a more task-constrained configuration that necessarily includes maintenance but additionally recruits specialized operations (e.g., monitoring, transformation, replacement). Under this framing, the ability-based account predicts stronger transfer from span to updating because maintenance training should strengthen a broadly applicable component on which updating depends. In contrast, the resource-based account predicts stronger updating to span transfer because higher-demand updating training should recruit a superset of resources sufficient for span performance. Finally, similarity-based accounts, without a priori auxiliary assumptions, predict no systematic directional advantage because transfer is expected to depend primarily on shared substrates/processes (e.g., frontoparietal network engagement; Nee et al., 2013; Rottschy et al., 2012), and such overlap is inherently nondirectional (Fig. 1B).

### Factors modulating WM transfer advantages

Directional transfer asymmetry, if present, may also vary across WM training contexts. Variables known to moderate overall transfer magnitude may also influence directionality, motivating moderator analyses. The first candidate is training dosage, although the evidence remains mixed: early meta-analytic work reported positive associations between training amount and transfer effects (Weicker et al., 2016), whereas later syntheses reported little or no moderation by total training hours (Soveri et al., 2017a), or effects that were largely confined to the nearest transfer outcomes (You et al., 2021). Given this controversy, dosage is treated here as an empirical moderator of directionality. A second moderator concerns developmental stage. Children and adolescents – those undergoing neurocognitive development – often show larger or more efficient transfer (Liu et al., 2023; Zhao et al., 2018), which is consistent with greater plasticity and/or greater headroom for improvement. Although headroom accounts are frequently linked to lower baseline performance in younger samples, the relationship between baseline and training-related gains or transfer is inconsistent across studies (Jaeggi et al., 2008; Wiemers et al., 2019; Zhu et al., 2017). Moreover, baseline measures are not consistently reported or readily comparable across studies. Accordingly, developmental stage (e.g., non-adults vs. adults) offers a more practical moderator that can be evaluated in meta-analytic syntheses. A third candidate is the stimulus domain (verbal vs. nonverbal), which is consistent with domain-specific WM subsystems (Baddeley, 1992; Baddeley & Hitch, 1974); however, evidence remains unclear. Some scholars argue that the novelty of visuospatial materials facilitates new learning and transfer (Gathercole et al., 2019; Norris et al., 2019), whereas competing evidence suggests stronger transfer following verbal WM training (Ni et al., 2023), which is potentially linked to more abstract symbolic operations. Notably, the stimulus domain here is usually classified on the basis of the representational nature of the memoranda instead of the representation modality. Taken together, these moderators are practically important, yet it remains unclear whether – and how – they systematically influence transfer directionality, highlighting the need for a systematic meta-analytic test of moderation patterns.

### The present meta-analysis

In summary, the existence and directional bias of transfer asymmetry in WM training remain empirically contested, and competing frameworks generate different predictions about cross-task transfer advantages. To address this ambiguity, the present meta-analysis contrasts transfer efficacy between two core WM paradigms: span tasks and updating tasks. Specifically, we aim to (1) test whether transfer between span and updating paradigms is reliably asymmetric by quantifying the direction of any asymmetry (updating→span vs. span→updating); (2) examine whether any observed transfer advantage generalizes to non-WM outcomes as an additional test, where if the asymmetry reflects a broader property of training rather than a narrow relation between two WM paradigms, the transfer advantage should also be detectable in more distal non-WM outcomes;^1^ and (3) test whether training dosage, developmental stage, and stimulus domain systematically moderate transfer directionality.

## Method

Before conducting the initial analyses, we uploaded an a priori analysis-plan document to the Open Science Framework (OSF). This document specified the initial hypotheses, inclusion criteria, and analytic procedures. During revision, we uploaded a separate additional analysis-plan document describing the analyses added in response to reviewer feedback. Both documents are publicly available as files within the same OSF project (https://doi.org/10.17605/OSF.IO/2S64Y).^2^

### Literature search and inclusion criteria

We conducted a systematic search across both English- and Chinese-language databases. The English-language databases included Web of Science, Elsevier, ProQuest, and Google Scholar, while the Chinese-language databases included CNKI (China National Knowledge Infrastructure) and Wanfang Data. The search covered all relevant studies published up to September 30, 2024. For English-language literature, the following Boolean string was used: (“simple span” OR “complex span” OR updat* OR “n-back” OR “working memory” OR “short* memory”) AND (training OR transfer). For Chinese-language literature, the search terms were specified in simplified Chinese as follows: (“简单广度” OR “复杂广度” OR “更新” OR “短时记忆” OR “工作记忆”) AND (“训练” OR “迁移”). To ensure comprehensive coverage, we further examined reference lists of seminal meta-analyses and systematic reviews (Constantinidis & Klingberg, 2016; Sala et al., 2019a; Sala & Gobet, 2020; Soveri et al., 2017a). Moreover, citation tracking was conducted via Google Scholar on two foundational studies (Dahlin et al., 2008a; Jaeggi et al., 2008). In addition, we contacted the corresponding authors of potentially relevant articles to obtain unpublished or incomplete data. Furthermore, one preprint and one unpublished study conducted by the authors were also included.

Studies were included on the basis of the following criteria:

1. Each study included at least one adaptive WM training group and one control group, with both pre-training and post-training transfer tests in each group. Adaptive procedures ensured that task difficulties in span and updating trainings were calibrated.
2. The training tasks included either span tasks or updating tasks. Span tasks included simple span (e.g., forward/backward recall, delay estimation, and change detection) and complex span (e.g., operation span, categorization span, and symmetry span) paradigms. Updating tasks included n-back and running memory. Although backward and complex span tasks involve executive components beyond simple storage, prior latent-variable evidence indicates that they load on a common span construct (Engle et al., 1999; Redick & Lindsey, 2013), and meta-analytic conventions have consistently classified them within the span domain rather than with updating paradigms (Sala & Gobet, 2020). Transfer effects were estimated by at least one untrained WM task. For instance, if training utilized span tasks, transfer assessment ​required at least one updating task.
3. Only healthy individuals were included; studies involving clinical populations (e.g., individuals with cognitive impairments, depression, attention-deficit hyperactivity disorder) were excluded to minimize confounding effects from individual differences.
4. Duplicate datasets from multiple publications were excluded.

**Fig. 2:**
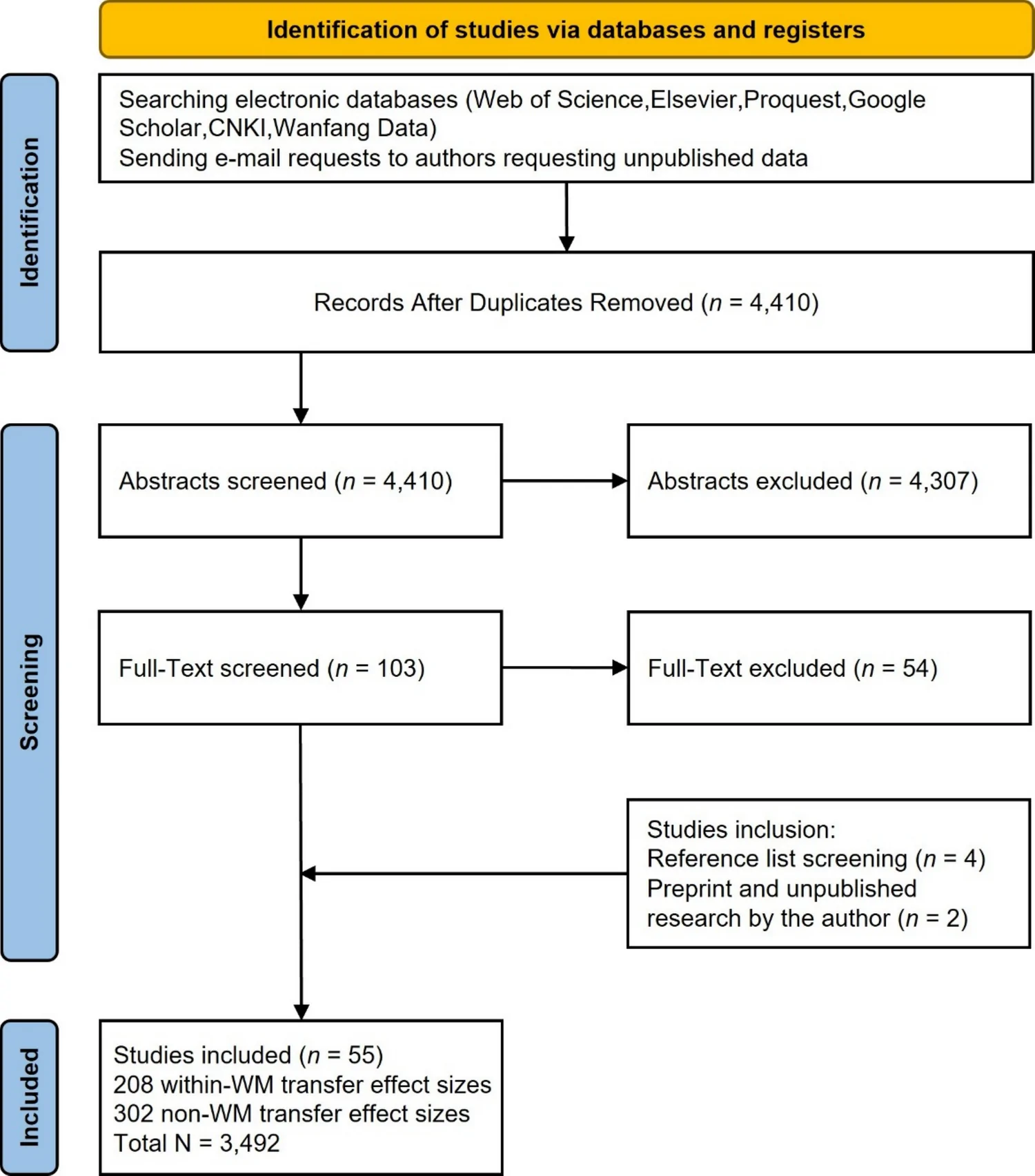
PRISMA (Preferred Reporting Items for Systematic Reviews and Meta-Analyses) flow outlining the study selection. WM = working memory

In summary, 55 independent studies, comprising 208 effect sizes and a total of 3,492 participants, were included in the primary within-WM meta-analysis. In addition, for the analysis of transfer beyond the WM domain, 302 non-WM effect sizes were identified from the included studies.^‡^ Detailed study characteristics and effect-size coding are reported in Supplementary Table S1 (Online Supplementary Material (OSM)) for the within-WM analysis and in Supplementary Table S2 (OSM) for analyses of transfer to non-WM outcomes. The PRISMA flowchart of this process is shown in Fig. 2.

### Data coding procedure and reliability

We developed a standardized coding procedure for extracting information from all included studies. Following this procedure, the first author coded all the studies, and a second author independently coded them again. Intercoder reliability was exceptionally high, with an overall agreement rate of 98%. Any discrepancies were resolved through discussion in consultation with the corresponding author until consensus was reached.

### Effect size calculation

Given the small sample sizes in the included studies, we employed Hedges’ *g*, a bias-corrected version of Cohen’s *d*, to quantify the effect size of training transfer (Hedges, 1981). Hedges’ *g* was calculated using the following formula (Morris, 2008; Schmidt & Hunter, 2015):

$$g = \frac{({M}_{{e}_{-}post}-{M}_{{e}_{-}pre})-({M}_{{c}_{-}post}-{M}_{{c}_{-}pre})}{S{D}_{poole{d}_{-}pre}}\times \left(1-\frac{3}{(4\times N)-9}\right)$$

where *M*_e_post_ and *M*_e_pre_ denote the mean performance of the training group at the post-training test and pre-training test, respectively. *M*_c_post_ and *M*_c_pre_ represent the mean performance of the control group at the corresponding time points, *SD*_pooled_pre_ is the pooled standard deviation (*SD*) of pre-training performance across both groups. *SD*_pooled_pre_ is estimated since the *SD*s of the pre-training and post-training tests within groups are not independent (Morris, 2008; Schmidt & Hunter, 2015). *N* is the total sample size. A positive Hedges’ *g* value indicates superior transfer efficiency in the training group versus the control group. Following conventional standards (Cohen, 1988), Hedges’ *g* values of 0.2, 0.5, and 0.8 were interpreted as small, medium, and large effects, respectively. Effect sizes with absolute values exceeding 3.0 were excluded as outliers (Sala et al., 2019a).

The sampling error variance for Hedges’ *g* was computed using the following formula:

$$Va{r}_{g}=\left(\frac{{N}_{e}-1}{{N}_{e}-3}\times \left(\frac{2\times (1-r)}{{r}_{xx}}+\frac{{d}_{e}^{2}}{2}\times \frac{{N}_{e}}{{N}_{e}-1}\right)\times \frac{1}{{N}_{e}}+\frac{{N}_{c}-1}{{N}_{c}-3}\times \left(\frac{2\times (1-r)}{{r}_{xx}}+\frac{{d}_{c}^{2}}{2}\times \frac{{N}_{c}}{{N}_{c}-1}\right)\times \frac{1}{{N}_{c}}\right)\times {\left(1-\frac{3}{(4\times N)-9}\right)}^{2}$$

where r_xx_ is the test-retest reliability coefficient of the transfer task; r is the correlation between task performance in the pre-training and post-training tests; *N* is the total sample size; *N*_e_ and *N*_c_ denote the sample sizes of the training and control groups, respectively; and d_e_ and d_c_ are the standardized mean differences within the training/control group, respectively. For studies lacking reported *r*_xx_ or *r* values, we adopted *r*_xx_ = *r* = 0.65 following previous WM-training meta-analytic practice (Sala et al., 2019a) and the psychometric meta-analytic approach (Schmidt & Hunter, 2015).

## Moderators

To evaluate potential asymmetric transfer effects between span and updating tasks, transfer direction was examined as the primary moderator in the present meta-analysis. In addition, we conducted an extension analysis to examine whether any directional transfer advantage observed within the WM domain also generalized to non-WM outcomes. Furthermore, three secondary moderators were investigated to assess their influence on transfer asymmetry: training dosage, participant age, and training stimulus domain.

1. **Transfer direction**: Both training and transfer tasks were explicitly labeled as either “span tasks” or “updating tasks.” Transfer direction was then coded at the level of each effect size on the basis of the specific combination of training and transfer tasks. Accordingly, effect sizes were categorized into two groups: span-to-updating (reflecting transfer from span training to untrained updating tasks) and updating-to-span (reflecting transfer from updating training to untrained span tasks).
2. **Transfer to the non-WM domain:**^‡^ To examine whether the observed transfer advantage extended beyond the WM domain, we additionally coded non-WM outcomes from studies that included such measures. These outcomes comprised processing speed, inhibition, shifting, mathematical ability, intelligence-related measures, and academic achievement. The corresponding effect sizes were extracted and analyzed separately from the primary within-WM analysis to test whether any directional transfer advantage observed within the WM domain was also evident for non-WM outcomes. In these analyses, transfer direction was coded according to the training task as either updating-to-other (reflecting transfer from updating training to non-WM outcomes) or span-to-other (reflecting transfer from span training to non-WM outcomes). Because intelligence-related outcomes were both theoretically important and frequently reported, we also conducted a more restricted supplementary analysis limited to this subset of non-WM measures.
3. **Training dosage:** The training dose was calculated as the product of the single-session duration and the total session count, and the studies were categorized into high- and low-training-dose groups. To minimize arbitrary categorization, we conducted a quartile analysis of the training dose and used the results as empirically derived cutoffs: Q1 = 5 h (25th percentile, the lower quartile boundary); Q2 = 7 h (50th percentile, the median); and Q3 = 10 h (75th percentile, the higher quartile boundary). For studies reporting imprecise session durations (e.g., “20–25 min”), the minimum value was used to calculate the total training dose (Soveri et al., 2017a).
4. **Developmental stage**: Participants were categorized as non-adults (< 18 years) and adults (≥ 18 years) on the basis of the mean age reported in each study. Mean age was used to ensure consistent classification across studies, particularly when explicit sample labels (e.g., “children” or “adults”) were not provided. Although age-related differences within adulthood (e.g., younger vs. older adults) are potentially theoretically relevant, a more fine-grained classification was not implemented because of limited and unbalanced data across transfer directions.
5. **Training domain**: Stimuli used in training tasks were classified on the basis of the representational nature of the memoranda, broadly following the distinctions proposed in multicomponent models of WM (Baddeley, 1992, 2012), into three categories: (a) verbal stimuli (e.g., letters, digits, words, and sentences; as a coding rule, these were coded as verbal regardless of representation modality); (b) nonverbal/visuospatial stimuli (e.g., spatial locations, objects, colors, and pictorial materials); and (c) combined or integrated stimuli involving both verbal and nonverbal components within the same task. For moderator analyses, we focused on studies in which the primary representational format could be clearly classified as either verbal or nonverbal. Tasks involving combined representations (e.g., dual n-back tasks or multimodal stimuli) were excluded from this analysis to prevent confounding effects arising from overlapping stimulus types. In a supplementary moderator analysis, we further examined stimulus-domain congruence by classifying transfer effects as within-domain or across-domain according to whether the training and transfer tasks involved the same stimulus domain (e.g., verbal-to-verbal) or different stimulus domains (e.g., verbal-to-nonverbal).^‡^

### Modeling approach

All the following statistical analyses were conducted in R (version 4.4.2; R Core Team, 2024) using the “rma.mv” function from the *metafor* package (Viechtbauer, 2010). Given that 42 of the 55 included studies contributed multiple effect sizes – violating the independence assumption of conventional meta-analysis – we adopted a three-level random-effects meta-analysis model (Van den Noortgate et al., 2013). This model accounts for three sources of variance: the sampling variance of individual effect sizes (Level 1), the variance among effect sizes within the same study (Level 2), and the variance between studies (Level 3) (Shi et al., 2021; Van den Noortgate et al., 2013). Unlike traditional approaches (e.g., averaging effect sizes within studies or selecting a single effect size per study), the three-level model incorporates all available data, improving statistical power and reducing the risk of Type I error (Van den Noortgate et al., 2013). In the current study, we first fitted an unconditional three-level model (without moderators) and estimated the overall transfer effect size between the updating and span tasks. The three-level model is formally expressed as follows:

$${\widehat{\theta }}_{ij}=\mu +{\zeta}_{(2)ij}+{\zeta}_{(3)j}+{\epsilon}_{ij}$$

where ${\widehat{\theta }}_{ij}$ represents the observed effect size *i* in study *j*; *μ* represents the overall average effect; *ζ*_*(2)ij*_ represents the level 2 variance (within-study heterogeneity), *ζ*_*(3)j*_ represents the level 3 variance (between-study heterogeneity), and *ϵ*_*ij*_ is the sampling variance of the effect size *i* in the study* j*. If significant heterogeneity was detected at level 2 or level 3 (*p* < 0.05), we conducted further moderator analysis (Raudenbush et al., 1991).

Then, we introduced the transfer direction as the primary moderator via a three-level mixed-effects meta-analysis model:

$${\widehat{\theta }}_{ij}=\theta +{\beta {x}_{i}+\zeta }_{(2)ij}+{\zeta}_{(3)j}+{\epsilon}_{ij}$$

where *θ* is the intercept and *β* is the regression coefficient associated with moderator *x*_*i*_, with a dummy-coded transfer direction: 0 denotes transfer from updating training to span tasks, and 1 denotes transfer from span training to updating tasks. Specifically, in this model, we evaluated the effect sizes for both transfer directions and the moderating effect of transfer direction was estimated by the differences between them.

Furthermore, we performed subgroup analyses for three secondary moderators: training dosage (low vs. high), developmental stage (non-adults vs. adults) and training stimulus domain (nonverbal vs. verbal). Within each subgroup defined by these moderators, we refitted separate three-level mixed-effects models and reintroduced the direction of transfer as a moderator. This approach allowed us to quantify the consistency of directionality effects across subgroups and identify potential contextual dependencies in transfer patterns.

### Sensitivity analysis

We conducted an influential case analysis to evaluate whether individual effect sizes disproportionately affected our meta-analytic estimates (Viechtbauer & Cheung, 2010). This analysis employed a leave-one-out procedure, iteratively excluding each effect size to quantify its impact on two key parameters: meta-analytic mean effect (*g*) and between-effect heterogeneity (τ^2^). Influential cases were subsequently identified on the basis of four classical regression diagnostics, including DFFITS, Cook’s distance, DFBETAS, and hat values. An effect size was considered influential if it met at least one of the following criteria:

- (1) |DFFITS| > 3 × √(p/(k - p))
- (2) Cook’s distance in the upper 50% of a χ^2^ distribution with p degrees of freedom
- (3) Hat value > 3 × (p/k)
- (4) |DFBETAS| > 1

where p is the number of model coefficients and k is the number of effect sizes. The stability of our findings was confirmed by ​recalculating the meta-analytic models after the removal of influential cases; the results remained stable, indicating that the conclusions were not unduly driven by outliers. The analysis was performed using the “influence()” function in the *metafor* R package.

### Publication bias

To determine the risk of publication bias, we first generated a funnel plot by plotting individual study effect sizes (x-axis) against their standard errors (y-axis). In the absence of publication bias, studies should be symmetrically distributed around the pooled effect estimate, forming an inverted funnel shape where higher-precision studies cluster near the mean while lower-precision studies scatter broadly at the base (Sterne et al., 2011). We then conducted ​Egger’s regression test, which quantifies funnel plot asymmetry by regressing standardized effect sizes (effect size divided by standard error) against precision. A nonsignificant intercept (*p* >.05) indicates no systematic asymmetry and low publication bias risk (Egger et al., 1997). Additionally, we computed the fail-safe number (*N*_fs_), which estimates the minimum number of unpublished null-result studies required to overturn the statistical significance of the current meta-analytic effect. Following the established criteria, we considered the findings potentially vulnerable to publication bias if *N*_fs_ < 5k + 10 (where k is the number of included studies) (Rothstein et al., 2005).

## Results

### Overall cross-paradigm near transfer effect

A three-level meta-analysis model revealed a significant cross-paradigm transfer effect between updating and span tasks (Hedges’ *g* = 0.146, 95% confidence interval (CI) [.077,.214], *p* <.001; Appendix A in the OSM lists all effect sizes). This transfer effect demonstrated no significant within-study heterogeneity (ω^2^ = 0.000, χ^2^(1) = 0.000, *p* = 1.000) but significant between-studies heterogeneity (τ^2^ = 0.023, χ^2^(1) = 23.699, *p* <.001). These results confirmed a significant near-transfer effect within the WM domain. Critically, the substantial between-studies heterogeneity underscored the need for further moderator analyses.

### Primary moderator analysis: Directional asymmetry in within-WM transfer

Primary moderator analysis demonstrated an asymmetric transfer pattern: updating training significantly transferred to span tasks (*g* = 0.176, 95% CI [.104,.249], *p* <.001), whereas span training showed no significant transfer to updating tasks (*g* = 0.048, 95% CI [-.078,.173], *p* =.453). The transfer effect from updating-to-span was marginally larger than that from span-to-updating (*F*(1, 202) = 3.259, *p* =.073; see Table 1).

**Table 1:** Primary moderator analysis of within-working memory transfer

Moderator	*m*	*k*	*g*	*SE*	*p*	95% CI	*F*	*p*
Direction	54	204					3.259	.073^+^
updating to span	42	161	0.176^***^	.037	<.001	[.104,.249]		
span to updating	16	43	0.048	.064	.453	[-.078,.173]		

*m* = number of studies; *k* = number of effect sizes; *g* = estimated effect size;

^+^ *p* <.10, ^*^ *p* <.05, ^**^ *p* <.01, ^***^ *p* <.001

To address potential confounding from task subtypes, we conducted follow-up analyses stratifying tasks into: (a) dual n-back (requiring simultaneous monitoring of two sensory modalities) versus single n-back paradigms (Küper & Karbach, 2016), and (b) simple span versus complex span tasks (requiring completion of an interleaved nonmemory task) (Harrison et al., 2013). These analyses consistently replicated the asymmetric transfer pattern (see OSM Table S3), excluding the possibility that the asymmetry was caused by subtype variation. Therefore, we aggregated all n-back tasks under a unified “updating task” category and all span-based measures under a “span task” category, thereby increasing the statistical power for detecting secondary moderator effects.

### Additional analysis of non-WM transfer outcomes

As an extension of the primary within-WM directional analysis, we examined whether the observed advantage of updating training also generalized to non-WM outcomes. When transfer direction was entered as the moderator, updating training showed a significant transfer effect to non-WM domains (*g* = 0.120, 95% CI [0.048, 0.192], *p* =.001), whereas span training did not (*g* = 0.008, 95% CI [−0.102, 0.118], *p* =.885). The difference between updating-based and span-based far transfer was marginally significant (*F*(1, 298) = 3.174, *p* =.076; see Table 2). A more restricted supplementary analysis focusing on intelligence-related measures yielded a consistent pattern (see OSM Table S4).

**Table 2:** Primary moderator analysis of transfer to non-working memory outcomes

Moderator	*m*	*k*	*g*	*SE*	*p*	95% CI	*F*	*p*
Direction	43	300					3.174	.076^+^
updating to other	35	204	0.120^**^	.037	.001	[0.048,.192]		
span to other	11	96	0.008	.056	.885	[−0.102,.118]		

Note:* m* = number of studies; *k* = number of effect sizes; *g* = estimated effect size;

^+^ *p* <.10, ^*^ *p* <.05, ^**^ *p* <.01, ^***^ *p* <.001

### Secondary moderator analysis: Moderators of the within-WM transfer asymmetry

#### **Training dosage**

When training dosage was classified on the basis of the lower quartile boundary (5 h), the low-dosage group exhibited a significant asymmetric transfer pattern: updating training yielded a significant transfer effect to span tasks (*g* = 0.200, 95% CI [.057,.342], *p* =.007), whereas span training did not transfer to updating tasks (*g* = −0.034, 95% CI [-.200,.133], *p* =.686). Critically, the transfer effect from updating to span was significantly greater than that from span to updating (*F*(1, 57) = 4.551, *p* =.037). In contrast, in the high-dosage group, although only updating training retained significant transfer (*g* = 0.178, 95% CI [.094,.262], *p* <.001), the difference between the two transfer directions was not significant (*F*(1, 143) = 0.490, *p* =.485; see Table 3). Notably, no significant differences in effect sizes were observed between the low- and high-dose groups for either transfer direction (*Fs* < 1.563, *ps* >.218). The asymmetric transfer patterns persisted when dosage thresholds were increased to 7 h and 10 h, although the statistical significance was attenuated (see Appendix E, Table S5 (OSM)). Follow-up moderator analyses were conducted in finer training dosage ranges (less than 5 h, 5–7 h, 7–10 h, and greater than 10 h), and the results suggested that this attenuation may stem from increased standard error of the span-to-updating transfer estimates with prolonged training duration (see Appendix E, Table S6 (OSM)).

**Table 3:** Secondary moderator analysis of within-working memory transfer asymmetry

Analysis type	Moderator	*m*	*k*	*g*	*SE*	*P*	95% CI	*F*	*P*
Training dosage									
low-dosage (hour ≤ 5 h)	Direction	20	59					4.551	.037^*^
	updating to span	14	35	0.200^**^	.071	.007	[.057,.342]		
	span to updating	6	24	−0.034	.083	.686	[-.200,.133]		
high-dosage (hour > 5 h)	Direction	38	145					0.490	.485
	updating to span	32	126	0.178^***^	.042	<.001	[.094,.262]		
	span to updating	10	19	0.113	.087	.196	[-.059,.285]		
Developmental stage									
non-adults	Direction	9	47					18.19	<.001^***^
	updating to span	5	26	0.362^***^	.076	<.001	[.209,.516]		
	span to updating	5	21	−0.056	.062	.370	[-.180,.068]		
adults	Direction	44	152					0.695	.406
	updating to span	36	130	0.157^***^	.040	<.001	[.078,.236]		
	span to updating	11	22	0.087	.078	.271	[-.068,.241]		
Training domain									
verbal stimuli	Direction	12	42					3.346	.075^+^
	updating to span	8	29	0.256^***^	.060	<.001	[.134,.378]		
	span to updating	4	13	0.063	.087	.471	[-.112,.238]		
nonverbal stimuli	Direction	16	55					1.446	.235
	updating to span	10	38	0.179	.114	.124	[-.051,.409]		
	span to updating	6	17	−0.042	.144	.771	[-.331,.246]		

*m* = number of studies; *k* = number of effect sizes; *g* = estimated effect size;

^+^ *p* <.10, ^*^ *p* <.05, ^**^ *p* <.01, ^***^ *p* <.001

#### **Developmental stage**

A significant asymmetric transfer pattern was observed in non-adults: updating training significantly transferred to span tasks (*g* = 0.362, 95% CI [.209,.516], *p* <.001), whereas span training yielded no significant transfer to updating tasks (*g* = −0.056, 95% CI [-.180,.068], *p* =.370). Moreover, the transfer effect size for updating training was significantly greater than that for span training (*F*(1, 45) = 18.19, *p* <.001). However, in adults, although a similar descriptive pattern was observed – updating training showed a significant transfer to span tasks (*g* = 0.157, 95% CI [.078,.236], *p* <.001), and span training did not transfer to updating (*g* = 0.087, 95% CI [-.068,.241], *p* =.271) – the asymmetry between transfer directions was not statistically supported in adults (*F*(1, 150) = 0.695, *p* =.406). This developmental divergence may stem from a quantitatively greater updating-to-span transfer effect in non-adults than in adults (*F*(1, 154) = 2.072, *p* =.152), whereas the span-to-updating transfer effect did not differ between the two populations (*F*(1, 41) = 1.498, *p* =.228). These findings support the hypothesis that cognitive/neural plasticity during developmental stages amplifies both the magnitude and the asymmetry of transfer effects. Given the limited and unbalanced data for older adults, a separate directional comparison within subgroups of older adults was not feasible. To ensure stability, sensitivity analyses excluding older adult samples were conducted and yielded virtually identical results, indicating that the adult pattern was not driven by the inclusion of older participants (see Table S7, OSM).^‡^

#### **Training domain**

In the verbal stimulus training group, updating training significantly transferred to span tasks (*g* = 0.256, 95% CI [.134,.378], *p* <.001), whereas span training showed no significant transfer effect (*g* = 0.063, 95% CI [-.112,.238], *p* =.471). The difference between the two transfer directions was marginally significant (*F*(1, 40) = 3.346, *p* =.075). In contrast, in the nonverbal stimulus group, neither updating-to-span transfer (*g* = 0.179, 95% CI [-.051,.409], *p* =.124) nor span-to-updating transfer (*g* = −0.042, 95% CI [-.331,.246], *p* =.771) reached statistical significance. No significant differences emerged for either transfer direction between the two stimulus groups (*F*s < 0.026, *ps* >.873), and we proposed that the asymmetry observed in the verbal training group may be attributable to its smaller standard error (verbal: *SE* =.060 vs. nonverbal: *SE* =.114). To further examine whether this pattern depended on stimulus-domain congruence between training and transfer tasks, we conducted a supplementary moderator analysis distinguishing within-domain from across-domain transfer. This analysis indicated that the overall asymmetric pattern was not materially changed by whether the training and transfer tasks shared the same stimulus domain (see Table S8, OSM).

### Sensitivity analysis

Sensitivity analysis confirmed the stability of the cross-paradigm transfer effects. After four influential cases were excluded, the cross-paradigm transfer effect between the updating and span tasks remained significant (*g* = 0.142, *SE* =.036, 95% CI [.071,.212], *m* = 53 studies, *k* = 200 effect sizes, *p* <.001). Critically, both the primary and the secondary moderator analyses demonstrated consistent asymmetric transfer patterns after the exclusion of influential cases (for detailed results, see Table S10, OSM).

### Publication bias

**Fig. 3:**
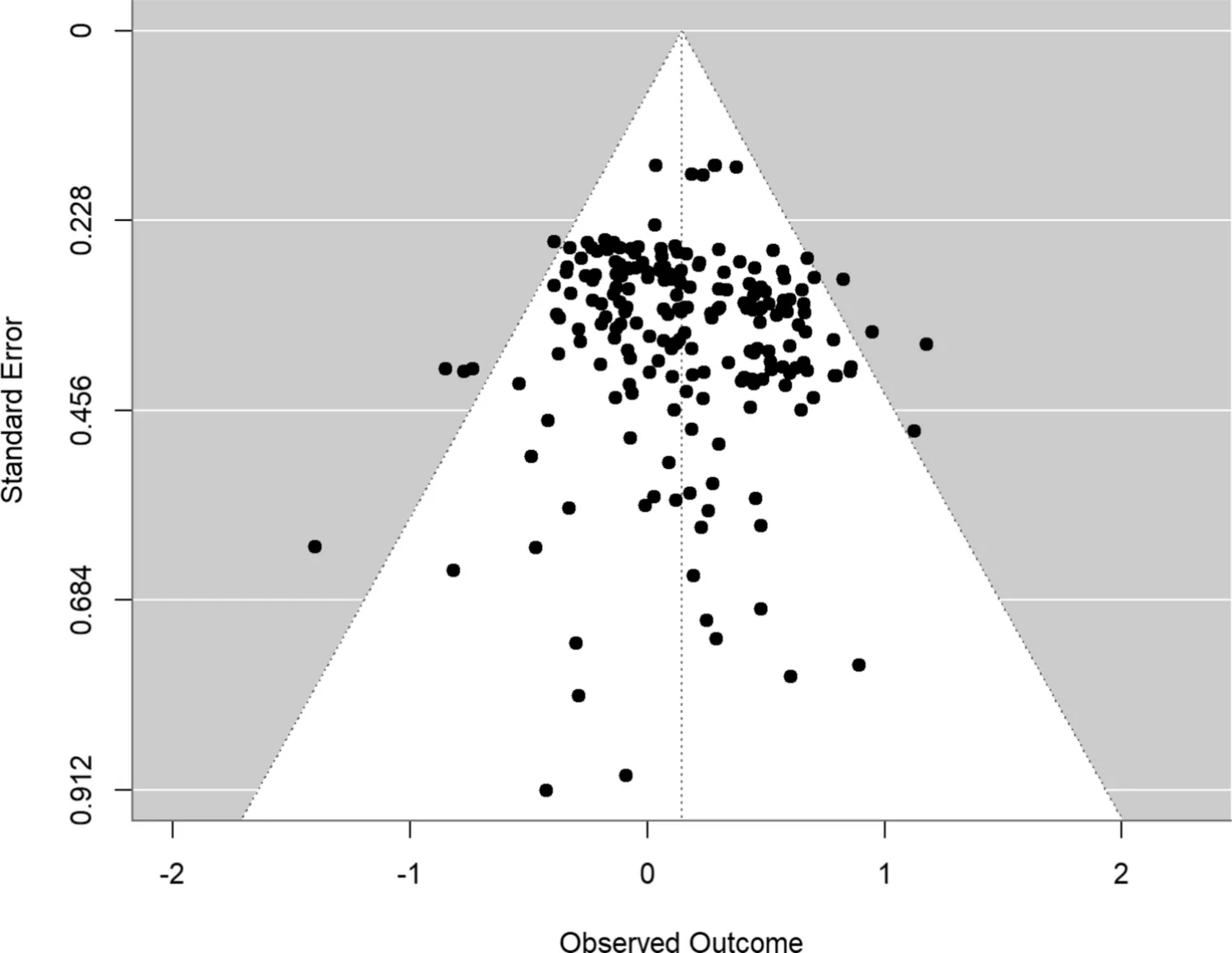
The funnel plot in the meta-analysis. Each dot denotes an effect size

The funnel plot exhibited notable symmetry (Fig. 3), and Egger’s regression test confirmed a nonsignificant intercept (*t*(202) = −0.087, *p* =.931), indicating that there was no systematic publication bias in the effect-size estimation. Furthermore, the fail-safe *N* of 2,816 far exceeded the critical threshold, reinforcing the reliability of the findings against potential unpublished studies. Taken together, these results provide no evidence of significant publication bias in the current meta-analysis.

## Discussion

This study provides the first meta-analytic evidence for directional asymmetry in WM training transfer. Specifically, updating-to-span transfer yielded significantly greater effects than span-to-updating transfer, supporting a resource-based transfer advantage hypothesis: training tasks that impose higher cognitive resource demands transfer more readily to tasks with lower demands than vice versa. Importantly, this asymmetry also extended to broader non-WM outcomes, suggesting that the transfer advantage of higher-demand training may generalize beyond paradigm-specific WM effects. Together, these findings extend traditional similarity-based accounts of WM transfer by indicating that transfer depends not only on the overlap between trained and transfer tasks but also on their relative cognitive resource demands. In addition, this asymmetry was most evident under lower training dosages, in non-adult samples, and in studies using verbal materials, indicating important boundary conditions for the proposed account.

### Directional asymmetry in WM transfer: Beyond similarity-based accounts

A central contribution of the present study is the direct testing of bi-directional transfer between two well-characterized WM paradigms. Within the WM domain, we observed a directional advantage of updating over span, and this asymmetry was evident across task subtypes, including simple and complex span tasks as well as single and dual n-back tasks. This pattern suggests that, beyond task similarity, the directional relation between paradigms plays a critical role in determining transfer. In other words, WM transfer appears to be shaped not only by whether two tasks overlap, but also by how their cognitive demands are structured relative to one another.

Unlike most previous meta-analyses, which examined transfer from one trained WM paradigm to a broad range of outcomes (Sala et al., 2019a; Soveri et al., 2017a), we focused exclusively on studies reporting span-to-updating or updating-to-span transfer. This design reduced conceptual heterogeneity and strengthened interpretability because the cognitive operations and neural correlates of both paradigms are relatively well characterized (Aben et al., 2012; Burgoyne et al., 2025). In addition, by restricting the analysis to adaptive training studies, we reduced the likelihood that the observed asymmetry reflected only differences in nominal task difficulty. Taken together, these design features support the conclusion that the observed asymmetry is theoretically meaningful: WM transfer appears to be shaped not only by task similarity but also by the relative level and organization of cognitive demand.

One possible explanation is that updating training, which requires the continuous monitoring and replacement of WM content, involves broader or more integrative processing than span training does, and therefore supports wider transfer. Evidence from other domains provides converging support for this resource-based account. Similar directional asymmetries have been reported in motor learning (Ikegami et al., 2010; Teixeira, 2000), raising the possibility that asymmetric transfer reflects a broader property of learning rather than a WM-specific phenomenon. This account has been interpreted in terms of differences in neural recruitment breadth, with training that engages broader networks transferring more readily to tasks relying on more localized or less integrative processing. For example, compared with nondominant-limb training, dominant-limb training results in more bilateral motor activation and stronger transfer (Teixeira, 2000), and a related pattern has been reported for discrete-to-rhythmic movement transfer (Ikegami et al., 2010; Schaal et al., 2004). However, such cross-domain parallels should be interpreted cautiously because similar behavioral asymmetries may arise from different forms of learning and plasticity across domains. Direct cross-domain research is needed to determine whether these patterns reflect a common principle or only analogous phenomena.

A related possibility is that more demanding training establishes more precise and stable representations, which in turn support transfer. Consistent with this view, auditory temporal discrimination training can transfer to visual tasks with lower precision demands, whereas the reverse pattern is not observed, presumably because the trained representations are insufficiently fine grained (Bratzke et al., 2012). Computational modeling is also consistent with this possibility, showing that models trained under higher-precision discrimination conditions generalize more broadly to lower-precision conditions than vice versa (Wenliang & Seitz, 2018). Consistent with this interpretation, transfer has been observed from high-precision visual perception training to relatively low-precision WM tasks (Jia et al., 2021), but not in the reverse direction (Cai et al., 2022). However, there have been few direct within-WM tests of this possibility, particularly within a single-study design.

A third possibility, motivated by recent WM evidence, is that the observed asymmetry reflects differences along a storage–manipulation continuum such that training requiring active manipulation promotes broader transfer than training focused primarily on maintenance does, resulting in the transfer asymmetry. Support for this possibility comes from evidence that cross-task generalization in frontoparietal systems is enhanced when tasks require active manipulation rather than simple maintenance, indicating that manipulations promote more abstract representations (Shi et al., 2025). This finding is also consistent with training findings showing that increasing manipulation complexity, such as in executive n-back tasks, leads to broader transfer outcomes (Cao et al., 2026). However, the present meta-analysis was not well positioned to test this account directly. Because only a small number of studies could be recoded using an alternative storage-to-manipulation versus manipulation-to-storage framework, our supplementary analysis revealed a similar trend but lacked enough statistical power to detect the transfer asymmetry (see Table S9, OSM).^‡^ Moreover, direct evidence within WM remains limited.

Importantly, these possible mechanisms are not mutually exclusive: broader neural recruitment, more precise representations, or more abstract representations may jointly contribute to asymmetric transfer. Determining whether and how these mechanisms interact to shape transfer direction will be important goals for future research.

### Resource-based transfer advantage as a broader training principle

Beyond the asymmetry observed between span and updating within WM, our findings suggest that the resource-based transfer advantage may generalize to broader transfer outcomes. Updating training showed broader transfer to non-WM outcomes, including intelligence-related measures, whereas span training showed little evidence of such effects. This pattern suggests that the observed asymmetry is not merely a paradigm-specific phenomenon within WM but may reflect a broader tendency for more cognitively demanding and integrative training to yield wider transfer than more circumscribed practice does.

These findings may help explain inconsistencies in the prior meta-analytic literature: meta-analyses that pooled heterogeneous WM paradigms, including both span- and updating-based training, often reported weaker or null far-transfer effects (e.g., Melby-Lervåg & Hulme, 2013; Sala & Gobet, 2020; Schwaighofer et al., 2015), whereas meta-analyses focusing specifically on updating/n-back training more often observed reliable, albeit small, transfer effects (e.g., Au et al., 2015; Soveri et al., 2017a; You et al., 2021). These converging patterns raise the possibility that asymmetric transfer reflects a broader tendency for training that recruits more extensive or integrative processing to transfer more readily than training that recruits less extensive or integrative processing. Thus, these findings have practical implications for training design, suggesting that interventions may be more effective when they incorporate demanding and integrative components rather than relying solely on routine or lower-demand practices. However, whether the processes underlying directional asymmetry within WM are the same as those supporting broader non-WM transfer remains unclear, and this question requires further investigation.

Importantly, the proposed resource-based transfer advantage may also have implications beyond the WM domain. Although the present meta-analysis used the updating-to-span contrast as a test case for this resource-based account, the account is not tied specifically to WM content. Rather, it concerns a broader resource-demand relation: transfer asymmetry may be expected when a high-demand task engages control processes that are also required by a lower-demand variant. From this perspective, the strongest rationale for generalization concerns other executive-function training domains, such as inhibition, set shifting and divided attention, where task variants may likewise differ systematically in control demands. This rationale is consistent with evidence that core executive functions are separable but partially overlapping (Friedman & Miyake, 2017; Miyake et al., 2000) and that demanding cognitive tasks can recruit overlapping domain-general control systems (Duncan, 2010; Fedorenko et al., 2013). Nevertheless, the applicability of this principle may be less clear in domains in which executive-control demands are less central, such as simple processing-speed training. At present, direct evidence for such asymmetries outside WM remains limited, warranting direct experimental and meta-analytic evaluation.

### Moderating conditions of the transfer advantage

Beyond the overall asymmetry, our moderator analyses revealed several conditions under which the transfer advantage was more pronounced. First, the transfer advantage was evident at low training doses, and increasing training duration was not associated with progressively greater transfer effects in either direction. This suggests that the transfer advantage does not simply scale with the amount of training, which is consistent with previous meta-analytic evidence showing no reliable dose-transfer relationship (Au et al., 2015; Soveri et al., 2017a). Au et al. further proposed that longer training may induce fatigue and reduce motivation, potentially offsetting any benefits of additional practice. However, this pattern should not be taken as direct evidence regarding fast versus slow learning phases in WM training (Ericson & Klingberg, 2023; Yamashita et al., 2015), because our training-dose categories were defined empirically from the distribution of training hours rather than from trajectory-based markers of learning. Although Ericson and Klingberg (2023) suggest that the transition to a slower learning phase may occur as early as 1.5 h in children, this estimate may not be generalizable to the populations and training protocols included here. Future studies that report detailed training trajectories will be important to clarify how transfer develops and whether the asymmetry changes across learning phases.

Second, developmental stage modulated the transfer asymmetry: Compared with adults, non-adults exhibited greater updating-to-span transfer, leading to more significant asymmetry. This pattern is consistent with evidence that children and adolescents may have greater transfer potential because their cognitive systems remain under development (Liu et al., 2023), as well as with prior meta-analytic findings of stronger cross-domain transfer from WM training in younger populations (Sala et al., 2019b). One possible explanation is that updating ability matures later than span ability does, leaving greater room for updating-based training to benefit young individuals (Ahmed et al., 2022; Skalaban et al., 2022). However, because developmental status and baseline capacity usually cannot be well disentangled in the meta-analytic data, this interpretation remains tentative.

Third, the training domain also shaped the observed transfer pattern. Studies using verbal materials showed clearer asymmetry than studies using nonverbal materials. One possible explanation is that verbal processing may facilitate more abstract or relational representations, whereas nonverbal materials may more often elicit concrete and feature-specific coding (Alfred & Kraemer, 2017). In the present dataset, some verbal materials also involved richer semantic processing (e.g., sentences), which may further support abstraction. This interpretation is broadly consistent with computational work highlighting the importance of feature-invariant representations for transfer (Johnston & Fusi, 2023; Yosinski et al., 2014). Notably, this pattern differs from evidence that visual novelty can facilitate within-paradigm transfer (Gathercole et al., 2019; Norris et al., 2019), perhaps because the paradigm-specific benefits associated with visual novelty may not support or even hinder cross-paradigm generalization. Overall, these moderator findings suggest that the transfer advantage is not only systematic but also context dependent.

### Limitations and future directions

Several directions for future research are important for further clarifying and extending the resource-based transfer advantage hypothesis. First, several methodological limitations of the present meta-analysis warrant consideration, including the greater number of studies examining updating-to-span than span-to-updating transfer, the relatively small number of studies in some moderator analyses, and the possibility that certain updating tasks were insufficiently sensitive to detect transfer from span training. Although the number of studies does not inherently determine effect size, such an imbalance may increase the risk of overestimating asymmetrical transfer (Rubio‐Aparicio et al., 2017). Future work under improved methodological conditions will therefore be important for replicating and refining the present findings. Second, because the present meta-analysis focused on healthy populations, it remains unclear whether the observed asymmetry can be generalized to clinical groups. This question is especially important given the heterogeneity of clinical conditions and emerging evidence that transfer patterns may vary across etiologies (Zhang et al., 2025). Third, future research should examine whether asymmetric transfer extends across modalities. Beyond the verbal–nonverbal distinction examined here, previous work has reported directional asymmetries across sensory modalities, such as olfactory-to-visual but not visual-to-olfactory transfer (Burke et al., 2025; Olofsson et al., 2020), suggesting that asymmetric transfer may reflect broader system-level constraints. Differences in how sensory pathways project to shared neural systems may contribute to such asymmetries and thus provide a complementary mechanism to the resource-based account proposed here. Finally, although the broader meta-analytic pattern in WM training is compatible with the resource-based interpretation, some findings suggest possible boundary conditions. Roque-Gutierrez and Ibbotson (2023), for example, reported an ability-based transfer advantage in the WM–linguistic domain, indicating that transfer asymmetry in higher-order cognitive training requires more study. In addition, differences in cognitive demand across domains (e.g., working memory vs. inhibition; Liu et al., 2023) are difficult to quantify using conventional behavioral measures, limiting direct tests of the resource-based account. Meanwhile, as discussed above, whether comparable resource-based asymmetries arise in other cognitive domains remains an important open question, particularly in other executive-function domains (e.g., divided attention, inhibition, and set shifting), but also in domains in which executive-control demands are less central (e.g., processing speed). Future studies incorporating neural or physiological indices may help quantify resource engagement across tasks and domains, thereby providing more precise tests of the resource-based account, including its boundary conditions and its generality beyond WM.

## Conclusion

In conclusion, the present meta-analysis provides the first systematic evidence for asymmetric transfer in WM training: updating-to-span transfer yields reliable gains, whereas span-to-updating transfer remains negligible. By showing that transfer depends not only on task similarity but also on relative cognitive demand, these findings extend traditional accounts of WM training transfer and support a resource-based perspective, according to which training on more demanding tasks transfers more effectively to less demanding tasks. This advantage further generalizes beyond WM, as updating training also produces reliable transfer to non-WM outcomes. Moreover, the asymmetry is especially pronounced under lower training doses, in younger samples, and with verbal materials, indicating important boundary conditions for transfer. Together, these findings advance the theoretical understanding of the transfer of cognitive training and provide practical guidance for designing more effective WM-based interventions in educational and clinical contexts.

## Supplementary Information

Below is the link to the electronic supplementary material.

Supplementary file1 (DOCX 139 KB)

## Data Availability

All study information and data for analysis are publicly available at the Open Science Framework website: https://doi.org/10.17605/OSF.IO/S8XN4

## Footnotes

1 Aim 2 was added during the revision process in response to reviewer feedback and was not part of the initial analysis plan. Throughout the article, analyses marked with a superscript double dagger (‡) were likewise added during revision in response to reviewer feedback.

2 Although OSF records the upload date and version history of each project file, these documents were not submitted through the formal OSF registration workflow and therefore should be understood as analysis-plan documents rather than frozen, read-only preregistrations.

## Code availability

The R code used for the analyses is publicly available at the Open Science Framework website: https://doi.org/10.17605/OSF.IO/S8XN4

## Declarations

### Open practices statement

The initial and additional analysis-plan documents are available on the Open Science Framework (https://doi.org/10.17605/OSF.IO/2S64Y). The data and analysis code for the current study are publicly available on the Open Science Framework (https://doi.org/10.17605/OSF.IO/S8XN4).
