# Supplementary material for: The asymmetry of working memory training transfer: A systematic review and meta-analysis
Source: Psychon Bull Rev. 2026 Sep 8;33(7):227. doi: 10.3758/s13423-026-02994-5 (PMC13553679; doi:10.3758/s13423-026-02994-5)
Supplement: Supplementary file 1 [file 13423_2026_2994_MOESM1_ESM.docx]

**Supplementary Appendix A. Included studies in the within-WM meta-analysis**

Table S1. Study characteristics for the within-WM meta-analysis

| **Study** | **id** | | **Training task** | **Training domain** | **Update type** | **Test task** | **Direction** | **Age** | **Hour** | **g** |
| --- | --- | --- | --- | --- | --- | --- | --- | --- | --- | --- |
| Ang et al. (2015) | 1 | | Cogmed | visual+verbal | none | Animal Updating Task | span_to_updating | non-adult | 18.750 | 0.186 |
| Ang et al. (2015) | 2 | | running span, keep track | visual | single | Letter Rotation Task | updating_to_span | non-adult | 12.500 | 0.451 |
| Ang et al. (2015) | 3 | | running span, keep track | visual | single | Block Recall task | updating_to_span | non-adult | 12.500 | -0.082 |
| Ang et al. (2015) | 4 | | running span, keep track | visual | single | Backward Letter Recall task | updating_to_span | non-adult | 12.500 | 0.430 |
| Anguera et al. (2012) | 5 | | dual n-back | visual+verbal | dual | Operation span | updating_to_span | adult | 1.875 | 0.545 |
| Booth et al. (2023) | 6 | | Distributed single n-back | visual+verbal | single | digit-span forward | updating_to_span | adult | 5.333 | -0.773 |
| Booth et al. (2023) | 7 | | Intensive single n-back | visual+verbal | single | digit-span forward | updating_to_span | adult | 5.333 | 0.135 |
| Booth et al. (2023) | 8 | | Distributed single n-back | visual+verbal | single | digit-span backward | updating_to_span | adult | 5.333 | 0.674 |
| Booth et al. (2023) | 9 | | Intensive single n-back | visual+verbal | single | digit-span backward | updating_to_span | adult | 5.333 | -0.139 |
| Bürki et al. (2014) | 10 | | verbal n-back | verbal | single | Reading span | updating_to_span | adult | 5.000 | 0.086 |
| Bürki et al. (2014) | 11 | | verbal n-back | verbal | single | Reading span | updating_to_span | adult | 5.000 | 0.143 |
| Cai et al. (2022) | 12 | | color delay estimation | visual | none | Spatial 2-Back Task | span_to_updating | adult | 12.000 | -0.046 |
| Chang (2019) | 13 | | single n-back | visual | single | Corsi’s Spatial Span | updating_to_span | non-adult | 10.000 | 0.578 |
| Chang (2019) | 14 | | single n-back | visual | single | Corsi’s Spatial Span | updating_to_span | non-adult | 10.000 | 0.826 |
| Chen (2021) | 15 | | running memory | visual+verbal | single | verbal simple span | updating_to_span | adult | 5.000 | 0.302 |
| Chen (2021) | 16 | | running memory | visual+verbal | single | visual simple span | updating_to_span | adult | 5.000 | -0.131 |
| Chen (2021) | 17 | | running memory | visual+verbal | single | verbal complex span | updating_to_span | adult | 5.000 | 0.658 |
| Chen (2021) | 18 | | running memory | visual+verbal | single | Symmetry Span | updating_to_span | adult | 5.000 | -0.196 |
| Chen (2021) | 19 | | running memory | visual+verbal | single | color change detection | updating_to_span | adult | 5.000 | -0.095 |
| Chooi & Thompson (2012) | 20 | | dual n-back task | visual+verbal | dual | Operation span | updating_to_span | adult | 4.000 | 0.185 |
| Chooi & Thompson (2012) | 21 | | dual n-back task | visual+verbal | dual | Operation span | updating_to_span | adult | 10.000 | 0.114 |
| Colom et al. (2013) | 22 | | visual n-back, verbal n-back, dual n-back | visual+verbal | complex | Reading span | updating_to_span | adult | 12.000 | 0.142 |
| Colom et al. (2013) | 23 | | visual n-back, verbal n-back, dual n-back | visual+verbal | complex | Computation span | updating_to_span | adult | 12.000 | -0.262 |
| Colom et al. (2013) | 24 | | visual n-back, verbal n-back, dual n-back | visual+verbal | complex | Dot matrix | updating_to_span | adult | 12.000 | 0.267 |
| Dahlin et al. (2008) | 25 | | running memory letters, keep track | visual+verbal | single | Dig-Span forward | updating_to_span | adult | 11.250 | 0.235 |
| Dahlin et al. (2008) | 26 | | running memory letters, keep track | visual+verbal | single | Dig-Span backward | updating_to_span | adult | 11.250 | -0.136 |
| Dahlin et al. (2008) | 27 | | running memory letters, keep track | visual+verbal | single | Computation span | updating_to_span | adult | 11.250 | -0.419 |
| Dahlin et al. (2008) | 28 | | running memory letters, keep track | visual+verbal | single | Dig-Span forward | updating_to_span | adult | 11.250 | -0.076 |
| Dahlin et al. (2008) | 29 | | running memory letters, keep track | visual+verbal | single | Dig-Span backward | updating_to_span | adult | 11.250 | 0.580 |
| Dahlin et al. (2008) | 30 | | running memory letters, keep track | visual+verbal | single | Computation span | updating_to_span | adult | 11.250 | 0.106 |
| Dahlin et al. (2008) | 31 | | running memory letters, keep track | visual+verbal | single | Dig-Span forward | updating_to_span | adult | 11.250 | 0.477 |
| Dahlin et al. (2008) | 32 | | running memory letters, keep track | visual+verbal | single | Dig-Span backward | updating_to_span | adult | 11.250 | -0.011 |
| Dahlin et al. (2008) | 33 | | running memory letters, keep track | visual+verbal | single | Computation span | updating_to_span | adult | 11.250 | 0.195 |
| Dahlin et al. (2008) | 34 | | running memory letters, keep track | visual+verbal | single | Dig-Span forward | updating_to_span | adult | 11.250 | -0.330 |
| Dahlin et al. (2008) | 35 | | running memory letters, keep track | visual+verbal | single | Dig-Span backward | updating_to_span | adult | 11.250 | 0.256 |
| Dahlin et al. (2008) | 36 | | running memory letters, keep track | visual+verbal | single | Computation span | updating_to_span | adult | 11.250 | 0.177 |
| Fellman et al. (2020) | 37 | | single n-back | verbal | single | Forward simple span with digits | updating_to_span | adult | 6.000 | 0.283 |
| Fellman et al. (2020) | 38 | | single n-back | verbal | single | Forward simple span with boxes | updating_to_span | adult | 6.000 | 0.033 |
| Fellman et al. (2020) | 39 | single n-back | | verbal | single | Backward simple span with digits | updating_to_span | adult | 6.000 | 0.375 |
| Fellman et al. (2020) | 40 | | single n-back | verbal | single | Backward simple span with boxes | updating_to_span | adult | 6.000 | 0.287 |
| Foster et al. (2017) | 41 | | running memory | visual+verbal | single | Reading span | updating_to_span | adult | 6.667 | 0.100 |
| Foster et al. (2017) | 42 | | running memory | visual+verbal | single | Rotation span | updating_to_span | adult | 6.667 | -0.291 |
| Foster et al. (2017) | 43 | | running memory | visual+verbal | single | Reading span | updating_to_span | adult | 6.667 | 0.511 |
| Foster et al. (2017) | 44 | | running memory | visual+verbal | single | Rotation span | updating_to_span | adult | 6.667 | -0.196 |
| Foster et al. (2017) | 45 | | operation span, symmetry span | visual+verbal | none | running span images | span_to_updating | adult | 6.667 | -0.113 |
| Foster et al. (2017) | 46 | | operation span, symmetry span | visual+verbal | none | running span arrows | span_to_updating | adult | 6.667 | 0.009 |
| Foster et al. (2017) | 47 | | operation span, symmetry span | visual+verbal | none | running span images | span_to_updating | adult | 6.667 | 0.157 |
| Foster et al. (2017) | 48 | | operation span, symmetry span | visual+verbal | none | running span arrows | span_to_updating | adult | 6.667 | 0.601 |
| Hadwin & Richards (2016) | 49 | | Cogmed | visual+verbal | none | verbal and spatial 2-back task | span_to_updating | non-adult | 12.500 | 0.439 |
| Harrison et al. (2013) | 50 | | operation-span, symmetry-span | visual+verbal | none | Running Letter Span | span_to_updating | adult | 15.000 | 3.478 |
| Harrison et al. (2013) | 51 | | operation-span, symmetry-span | visual+verbal | none | Running Matrix Span | span_to_updating | adult | 15.000 | 5.139 |
| Harrison et al. (2013) | 52 | | simple span | visual+verbal | none | letter running span | span_to_updating | adult | 15.000 | 4.219 |
| Harrison et al. (2013) | 53 | | simple span | visual+verbal | none | spatial running span | span_to_updating | adult | 15.000 | 4.170 |
| Heinzel et al. (2014) | 54 | | single n-back | verbal | single | Digit Span forward | updating_to_span | adult | 9.000 | 0.446 |
| Heinzel et al. (2014) | 55 | | single n-back | verbal | single | Digit Span backward | updating_to_span | adult | 9.000 | -0.064 |
| Heinzel et al. (2014) | 56 | | single n-back | verbal | single | Digit Span forward | updating_to_span | adult | 9.000 | 0.789 |
| Heinzel et al. (2014) | 57 | | single n-back | verbal | single | Digit Span backward | updating_to_span | adult | 9.000 | 0.598 |
| Holmes et al. (2019) | 58 | | Complex span | visual+verbal | none | verbal n-back | span_to_updating | adult | 10.000 | 0.236 |
| Holmes et al. (2019) | 59 | | Complex span | visual+verbal | none | visual n-back | span_to_updating | adult | 10.000 | -0.851 |
| Holmes et al. (2019) | 60 | | single n-back | visual+verbal | single | Complex span-verbal | updating_to_span | adult | 10.000 | -0.073 |
| Holmes et al. (2019) | 61 | | single n-back | visual+verbal | single | Complex span-visual | updating_to_span | adult | 10.000 | -0.738 |
| Jaeggi et al. (2009) | 62 | | dual n-back task | visual+verbal | dual | Digit span forward and backward | updating_to_span | NA | 7.917 | 0.567 |
| Jaeggi et al. (2009) | 63 | | dual n-back task | visual+verbal | dual | Change detection | updating_to_span | NA | 7.917 | 0.303 |
| Jaeggi et al. (2009) | 64 | | dual n-back task | visual+verbal | dual | Reading Span | updating_to_span | NA | 7.917 | -0.089 |
| Jaeggi et al. (2009) | 65 | | dual n-back task | visual+verbal | dual | Symmetry Span | updating_to_span | NA | 7.917 | -0.384 |
| Jaeggi et al. (2009) | 66 | | dual n-back task | visual+verbal | dual | Operation Span | updating_to_span | NA | 7.917 | 0.419 |
| Jaeggi et al. (2010) | 67 | | single n-back | visual | single | Operation span | updating_to_span | adult | 5.667 | -0.344 |
| Jaeggi et al. (2010) | 68 | | dual n-back task | visual+verbal | dual | Operation span | updating_to_span | adult | 5.667 | -0.278 |
| Jaeggi et al. (2008) | 69 | | dual n-back task | visual+verbal | dual | Reading span | updating_to_span | adult | 5.000 | -0.490 |
| Jaeggi et al. (2008) | 70 | | dual n-back task | visual+verbal | dual | Reading span | updating_to_span | adult | 7.083 | -0.302 |
| Jaeggi et al. (2008) | 71 | | dual n-back task | visual+verbal | dual | Reading span | updating_to_span | adult | 7.917 | 0.889 |
| Jaeggi et al. (2008) | 72 | | dual n-back task | visual+verbal | dual | Digit span forward | updating_to_span | adult | 3.333 | 0.602 |
| Jaeggi et al. (2008) | 73 | | dual n-back task | visual+verbal | dual | Digit span backward | updating_to_span | adult | 3.333 | 0.479 |
| Jaeggi et al. (2008) | 74 | | dual n-back task | visual+verbal | dual | Digit span forward and backward | updating_to_span | adult | 5.000 | 0.299 |
| Jaeggi et al. (2008) | 75 | | dual n-back task | visual+verbal | dual | Digit span forward and backward | updating_to_span | adult | 7.083 | 0.227 |
| Jaeggi et al. (2008) | 76 | | dual n-back task | visual+verbal | dual | Digit span forward | updating_to_span | adult | 7.917 | 0.248 |
| Jaeggi et al. (2008) | 77 | | dual n-back task | visual+verbal | dual | Visuospatial span forward | updating_to_span | adult | 7.917 | -0.819 |
| Jaeggi et al. (2008) | 78 | | dual n-back task | visual+verbal | dual | Visuospatial span backward | updating_to_span | adult | 7.917 | -0.470 |
| Jakoby et al. (2019) | 79 | | single n-back | visual | single | Operation span | updating_to_span | adult | 20.000 | 0.124 |
| Kang (2023) | 80 | | word simple span, word complex span, sentence complex span | verbal | none | 1-back | span_to_updating | non-adult | 3.750 | 0.674 |
| Kang (2023) | 81 | | word simple span, word complex span, sentence complex span | verbal | none | 2-back | span_to_updating | non-adult | 3.750 | -0.144 |
| Kang (2023) | 82 | | word simple span, word complex span, sentence complex span | verbal | none | 1-back | span_to_updating | non-adult | 3.750 | 0.058 |
| Kang (2023) | 83 | | word simple span, word complex span, sentence complex span | verbal | none | 2-back | span_to_updating | non-adult | 3.750 | -0.180 |
| Kang (2023) | 84 | | location simple span, 2 location complex span | visual | none | 1-back | span_to_updating | non-adult | 3.750 | -0.392 |
| Kang (2023) | 85 | | location simple span, 2 location complex span | visual | none | 2-back | span_to_updating | non-adult | 3.750 | -0.165 |
| Kang (2023) | 86 | | location simple span, 2 location complex span | visual | none | 1-back | span_to_updating | non-adult | 3.750 | -0.116 |
| Kang (2023) | 87 | | location simple span, 2 location complex span | visual | none | 2-back | span_to_updating | non-adult | 3.750 | -0.254 |
| Kang (2023) | 88 | | word simple span, word complex span, sentence complex span | verbal | none | 1-back | span_to_updating | non-adult | 3.750 | -0.232 |
| Kang (2023) | 89 | | word simple span, word complex span, sentence complex span | verbal | none | 2-back | span_to_updating | non-adult | 3.750 | 0.000 |
| Kang (2023) | 90 | | word simple span, word complex span, sentence complex span | verbal | none | 1-back | span_to_updating | non-adult | 3.750 | 0.179 |
| Kang (2023) | 91 | | word simple span, word complex span, sentence complex span | verbal | none | 2-back | span_to_updating | non-adult | 3.750 | 0.136 |
| Kang (2023) | 92 | | location simple span, location complex span | visual | none | 1-back | span_to_updating | non-adult | 3.750 | -0.110 |
| Kang (2023) | 93 | | location simple span, location complex span | visual | none | 2-back | span_to_updating | non-adult | 3.750 | 0.000 |
| Kang (2023) | 94 | | location simple span, location complex span | visual | none | 1-back | span_to_updating | non-adult | 3.750 | -0.219 |
| Kang (2023) | 95 | | location simple span, location complex span | visual | none | 2-back | span_to_updating | non-adult | 3.750 | -0.131 |
| Lawlor-Savage & Goghari (2016) | 96 | | dual n-back | visual+verbal | dual | Operation span | updating_to_span | adult | 10.417 | 0.094 |
| Lawlor-Savage & Goghari (2016) | 97 | | dual n-back | visual+verbal | dual | Dig-Span total | updating_to_span | adult | 10.417 | 0.073 |
| Lee (2014) | 98 | | animals complex span | visual | none | 2-back-animals (correct trials) | span_to_updating | non-adult | 1.500 | -0.393 |
| Lee (2014) | 99 | | animals complex span | visual | none | 2-back-animals (RT) | span_to_updating | non-adult | 1.500 | -0.080 |
| Lilienthal et al. (2013) | 100 | | dual n-back task | visual+verbal | dual | Operation span | updating_to_span | adult | 4.000 | 0.165 |
| Lilienthal et al. (2013) | 101 | | dual n-back task | visual+verbal | dual | Grid span | updating_to_span | adult | 4.000 | -0.074 |
| Lilienthal et al. (2013) | 102 | | dual n-back task | visual+verbal | dual | Cued recall span | updating_to_span | adult | 4.000 | 0.647 |
| Linares et al. (2018) | 103 | | Arithmetical updating task, Number size updating task | verbal | single | Operation span | updating_to_span | adult | 3.000 | 0.575 |
| Linares et al. (2019) | 104 | | single n-back | verbal | single | Operation span | updating_to_span | adult | 3.000 | 0.390 |
| Linares et al. (2019) | 105 | | single n-back | verbal | single | Operation span | updating_to_span | adult | 3.000 | -0.052 |
| Lindeløv et al. (2016) | 106 | | single n-back | visual+verbal | single | Operation span | updating_to_span | adult | 6.667 | 0.028 |
| Lindeløv et al. (2016) | 107 | | single n-back | visual+verbal | single | Digit Span backward | updating_to_span | adult | 6.667 | 0.274 |
| Lindeløv et al. (2016) | 108 | | single n-back | visual+verbal | single | letter-number sequencing | updating_to_span | adult | 6.667 | 0.456 |
| Lindsay (2012) | 109 | | single n-back | visual | single | Backwards Digits | updating_to_span | non-adult | 3.000 | -0.092 |
| Lindsay (2012) | 110 | | single n-back | visual | single | Corsi block span | updating_to_span | non-adult | 3.000 | -0.292 |
| Lindsay (2012) | 111 | | single n-back | visual | single | Color backward Span | updating_to_span | non-adult | 3.000 | -0.428 |
| Minear et al. (2016) | 112 | | Complex span | verbal | none | Verbal N-back | span_to_updating | adult | 10.000 | 0.117 |
| Minear et al. (2016) | 113 | | Complex span | verbal | none | Object N-back | span_to_updating | adult | 10.000 | 0.059 |
| Minear et al. (2016) | 114 | | spatial n-back | visual | single | Reading span | updating_to_span | adult | 6.667 | -0.234 |
| Minear et al. (2016) | 115 | | spatial n-back | visual | single | Operation span | updating_to_span | adult | 6.667 | 0.300 |
| Minear et al. (2016) | 116 | | spatial n-back | visual | single | Symmetry span | updating_to_span | adult | 6.667 | 0.127 |
| Minear et al. (2016) | 117 | | spatial n-back | visual | single | Rotation span | updating_to_span | adult | 6.667 | 0.528 |
| Minear et al. (2016) | 118 | | spatial n-back | visual | single | Alignment span | updating_to_span | adult | 6.667 | -0.169 |
| Minear et al. (2016) | 119 | | spatial n-back | visual | single | Letter-number span | updating_to_span | adult | 6.667 | -0.067 |
| Minear et al. (2016) | 120 | | spatial n-back | visual | single | Digit span forward | updating_to_span | adult | 6.667 | -0.328 |
| Minear et al. (2016) | 121 | | spatial n-back | visual | single | Letter Span | updating_to_span | adult | 6.667 | -0.040 |
| Minear et al. (2016) | 122 | | spatial n-back | visual | single | Circle Span | updating_to_span | adult | 6.667 | -0.214 |
| Minear et al. (2016) | 123 | | spatial n-back | visual | single | Arrow Span | updating_to_span | adult | 6.667 | 0.220 |
| Oelhafen et al. (2013) | 124 | | dual n-back task | visual+verbal | dual | Reading span | updating_to_span | adult | 5.833 | -0.131 |
| Pahor et al. (2022) | 125 | | n-back | visual+audio | dual | Simple Span | updating_to_span | adult | 6.667 | 0.234 |
| Pahor et al. (2022) | 126 | | n-back | visual+audio | dual | Complex Span | updating_to_span | adult | 6.667 | 0.188 |
| Pan et al. (2018) | 127 | | dual n-back | visual+verbal | dual | Corsi’s Spatial Span | updating_to_span | adult | 2.500 | 0.598 |
| Pergher et al. (2018) | 128 | | single n-back | visual | single | CORSI block‐tapping test | updating_to_span | adult | 5.000 | 0.291 |
| Pergher et al. (2018) | 129 | | single n-back | visual | single | CORSI block‐tapping test | updating_to_span | adult | 5.000 | -1.401 |
| Pugin et al. (2014) | 130 | | single n-back | visual | single | Letter-number sequencing-post | updating_to_span | non-adult | 8.000 | 0.190 |
| Pugin et al. (2014) | 131 | | single n-back | visual | single | Number-span-post | updating_to_span | non-adult | 8.000 | -0.540 |
| Qi (2024) | 132 | | delayed estimation task | visual | none | Location 2-back | span_to_updating | adult | 5.000 | -0.284 |
| Qi (2024) | 133 | | delayed estimation task | visual | none | Letter 2-back | span_to_updating | adult | 5.000 | -0.373 |
| Qi et al. (2024) | 134 | | delayed estimation task | visual | none | Location 2-back | span_to_updating | adult | 10.000 | 0.270 |
| Qi et al. (2024) | 135 | | delayed estimation task | visual | none | Color 2-back | span_to_updating | adult | 10.000 | 0.296 |
| Qi et al. (2024) | 136 | | delayed estimation task | visual | none | Letter 2-back | span_to_updating | adult | 10.000 | -0.174 |
| Rass et al. (2015) | 137 | | Cogmed | visual+verbal | none | N-back task (hit rate) | span_to_updating | adult | 18.750 | -0.049 |
| Rass et al. (2015) | 138 | | Cogmed | visual+verbal | none | N-back task (false alarm rate) | span_to_updating | adult | 18.750 | -0.079 |
| Rass et al. (2015) | 139 | | Cogmed | visual+verbal | none | N-back task d' (sensitivity) | span_to_updating | adult | 18.750 | 0.053 |
| Redick et al. (2013) | 140 | | dual n-back task | visual+verbal | dual | Symmetry Span | updating_to_span | adult | 11.667 | 0.335 |
| Redick et al. (2020) | 141 | | operation letter span | verbal | none | 3-back letter | span_to_updating | adult | 5.000 | 0.323 |
| Redick et al. (2020) | 142 | | operation letter span | verbal | none | 3-back matrix | span_to_updating | adult | 5.000 | -0.134 |
| Ripp et al. (2022) | 143 | | single n-back | visual+verbal | single | Corsi-block Tapping test | updating_to_span | adult | 10.667 | 0.054 |
| Ripp et al. (2022) | 144 | | single n-back | visual+verbal | single | digit-span forward | updating_to_span | adult | 10.667 | 0.703 |
| Ripp et al. (2022) | 145 | | single n-back | visual+verbal | single | digit-span backward | updating_to_span | adult | 10.667 | 0.098 |
| Schwarb et al. (2016) | 146 | | single n-back | visual+verbal | single | Operation span | updating_to_span | adult | 8.000 | 0.651 |
| Schwarb et al. (2016) | 147 | | single n-back | visual+verbal | single | symmetry span | updating_to_span | adult | 8.000 | 0.406 |
| Schwarb et al. (2016) | 148 | | single n-back | visual | single | Operation span | updating_to_span | adult | 5.333 | 0.475 |
| Schwarb et al. (2016) | 149 | | single n-back | visual | single | symmetry span | updating_to_span | adult | 5.333 | 0.635 |
| Schwarb et al. (2016) | 150 | | single n-back | verbal | single | Operation span | updating_to_span | adult | 5.333 | 0.446 |
| Schwarb et al. (2016) | 151 | | single n-back | verbal | single | symmetry span | updating_to_span | adult | 5.333 | 0.129 |
| Schwarb et al. (2016) | 152 | | single n-back | visual+verbal | single | Change detection shape_number | updating_to_span | adult | 8.000 | 0.477 |
| Schwarb et al. (2016) | 153 | | single n-back | visual+verbal | single | Change detection shape_resolution | updating_to_span | adult | 8.000 | 0.495 |
| Schwarb et al. (2016) | 154 | | single n-back | visual | single | Change detection shape_number | updating_to_span | adult | 5.333 | 0.947 |
| Schwarb et al. (2016) | 155 | | single n-back | visual | single | Change detection shape_resolution | updating_to_span | adult | 5.333 | 0.666 |
| Schwarb et al. (2016) | 156 | | single n-back | verbal | single | Change detection shape_number | updating_to_span | adult | 5.333 | 1.174 |
| Schwarb et al. (2016) | 157 | | single n-back | verbal | single | Change detection shape_resolution | updating_to_span | adult | 5.333 | 0.784 |
| Schwarb et al. (2016) | 158 | | single n-back | visual | single | Short-term recall task_number | updating_to_span | adult | 5.333 | 0.589 |
| Schwarb et al. (2016) | 159 | | single n-back | visual | single | Short-term recall task_resolution | updating_to_span | adult | 5.333 | 0.481 |
| Schwarb et al. (2016) | 160 | | single n-back | verbal | single | Short-term recall task_number | updating_to_span | adult | 5.333 | 0.433 |
| Schwarb et al. (2016) | 161 | | single n-back | verbal | single | Short-term recall task_resolution | updating_to_span | adult | 5.333 | 0.069 |
| Schweizer et al. (2011) | 162 | | dual n-back task | visual+verbal | dual | forward digit span | updating_to_span | adult | 6.667 | 0.395 |
| Schweizer et al. (2011) | 163 | | dual n-back task | visual+verbal | dual | forward digit span | updating_to_span | adult | 6.667 | 0.699 |
| Song et al. (2020) | 164 | | recall forward | visual | none | 2-back | span_to_updating | adult | 3.333 | 0.568 |
| Soveri et al. (2017) | 165 | | dual n-back task | visual+verbal | dual | Digit span forward | updating_to_span | adult | 5.000 | -0.117 |
| Soveri et al. (2017) | 166 | | dual n-back task | visual+verbal | dual | Digit span backward | updating_to_span | adult | 5.000 | -0.020 |
| Soveri et al. (2017) | 167 | | dual n-back task | visual+verbal | dual | Corsi block task forward | updating_to_span | adult | 5.000 | 0.215 |
| Soveri et al. (2017) | 168 | | dual n-back task | visual+verbal | dual | Corsi block task backward | updating_to_span | adult | 5.000 | -0.339 |
| Stepankova et al. (2014) | 169 | | verbal n-back | verbal | single | Digit span | updating_to_span | adult | 4.167 | 0.166 |
| Stepankova et al. (2014) | 170 | | verbal n-back | verbal | single | Letter Number Sequencing | updating_to_span | adult | 4.167 | 0.660 |
| Stepankova et al. (2014) | 171 | | verbal n-back | verbal | single | Digit span | updating_to_span | adult | 8.333 | 0.157 |
| Stepankova et al. (2014) | 172 | | verbal n-back | verbal | single | Letter Number Sequencing | updating_to_span | adult | 8.333 | 0.512 |
| Studer-Luethi & Meier (2021) | 173 | | dual n-back | visual+verbal | dual | Digit span forward | updating_to_span | adult | 6.667 | 0.133 |
| Tan et al. (2024) | 174 | | backward span, instructions activity | verbal | none | 2-back | span_to_updating | non-adult | 3.333 | 0.032 |
| Thompson et al. (2013) | 175 | | dual n-back task | visual+verbal | dual | Operation Span | updating_to_span | adult | 13.333 | 0.463 |
| Thompson et al. (2013) | 176 | | dual n-back task | visual+verbal | dual | Reading Span | updating_to_span | adult | 13.333 | 0.474 |
| Von Bastian & Eschen (2016) | 177 | | three complex span tasks | visual+verbal | none | Memory updating task | span_to_updating | adult | 10.000 | 0.164 |
| Von Bastian & Oberauer (2013) | 178 | | complex span | visual+verbal | none | Memory updating | span_to_updating | adult | 10.000 | 0.073 |
| Wang (2019) | 179 | | dual n-back | visual+verbal | dual | Digit span | updating_to_span | adult | 8.333 | 0.091 |
| Wang (2019) | 180 | | dual n-back | visual+verbal | dual | Change detection | updating_to_span | adult | 8.333 | 0.118 |
| Waris et al. (2015) | 181 | | Selective Updating training, figure updating, dual n-back | visual+verbal | complex | Digit span backward | updating_to_span | adult | 11.250 | 0.407 |
| Waris et al. (2015) | 182 | | Selective Updating training, figure updating, dual n-back | visual+verbal | complex | Corsi Block Task backward | updating_to_span | adult | 11.250 | 0.523 |
| Waris et al. (2015) | 183 | | Selective Updating training, figure updating, dual n-back | visual+verbal | complex | Digit span forward | updating_to_span | adult | 11.250 | 0.341 |
| Waris et al. (2015) | 184 | | Selective Updating training, figure updating, dual n-back | visual+verbal | complex | Corsi Block Task forward | updating_to_span | adult | 11.250 | 0.047 |
| Xiao (2019) | 185 | | dual n-back | visual+verbal | dual | Corsi block | updating_to_span | adult | 8.000 | 0.486 |
| Xiao (2019) | 186 | | dual n-back | visual+verbal | dual | letter span | updating_to_span | adult | 8.000 | -0.082 |
| Zając-Lamparska (2024) | 187 | | single n-back | verbal | single | Operation span | updating_to_span | adult | 9.000 | -0.231 |
| Zając-Lamparska (2024) | 188 | | single n-back | verbal | single | Dig-Span forward | updating_to_span | adult | 9.000 | -0.117 |
| Zając-Lamparska (2024) | 189 | | single n-back | verbal | single | Dig-Span backward | updating_to_span | adult | 9.000 | -0.141 |
| Zając-Lamparska (2024) | 190 | | single n-back | verbal | single | Operation span | updating_to_span | adult | 9.000 | -0.325 |
| Zając-Lamparska (2024) | 191 | | single n-back | verbal | single | Dig-Span forward | updating_to_span | adult | 9.000 | 0.447 |
| Zając-Lamparska (2024) | 192 | | single n-back | verbal | single | Dig-Span backward | updating_to_span | adult | 9.000 | 0.123 |
| Zhong (2011) | 193 | | single n-back | visual | single | Reading Span | updating_to_span | non-adult | 2.917 | 0.069 |
| Zhong (2011) | 194 | | dual n-back | visual+audio | dual | Reading Span | updating_to_span | non-adult | 2.917 | 0.519 |
| Zhong (2011) | 195 | | single n-back | visual | single | Reading Span | updating_to_span | non-adult | 5.833 | -0.197 |
| Zhong (2011) | 196 | | dual n-back | visual+audio | dual | Reading Span | updating_to_span | non-adult | 5.833 | -0.374 |
| Zhong (2011) | 197 | | single n-back | visual | single | Reading Span | updating_to_span | non-adult | 8.750 | 1.124 |
| Zhong (2011) | 198 | | dual n-back | visual+audio | dual | Reading Span | updating_to_span | non-adult | 8.750 | 0.571 |
| Zhong (2011) | 199 | | single n-back | visual | single | Reading Span | updating_to_span | non-adult | 11.667 | 0.010 |
| Zhong (2011) | 200 | | dual n-back | visual+audio | dual | Reading Span | updating_to_span | non-adult | 11.667 | 0.432 |
| Zhong (2011) | 201 | | single n-back | visual | single | Backward Digits | updating_to_span | non-adult | 2.917 | 0.852 |
| Zhong (2011) | 202 | | dual n-back | visual+audio | dual | Backward Digits | updating_to_span | non-adult | 2.917 | 0.859 |
| Zhong (2011) | 203 | | single n-back | visual | single | Backward Digits | updating_to_span | non-adult | 5.833 | 0.116 |
| Zhong (2011) | 204 | | dual n-back | visual+audio | dual | Backward Digits | updating_to_span | non-adult | 5.833 | 0.447 |
| Zhong (2011) | 205 | | single n-back | visual | single | Backward Digits | updating_to_span | non-adult | 8.750 | 0.626 |
| Zhong (2011) | 206 | | dual n-back | visual+audio | dual | Backward Digits | updating_to_span | non-adult | 8.750 | 0.432 |
| Zhong (2011) | 207 | | single n-back | visual | single | Backward Digits | updating_to_span | non-adult | 11.667 | 0.794 |
| Zhong (2011) | 208 | | dual n-back | visual+audio | dual | Backward Digits | updating_to_span | non-adult | 11.667 | 0.657 |

Note: Each row represents one effect size included in the within-WM meta-analysis. In some studies, multiple effect sizes were extracted for the same training-task and transfer-task combination because the original article reported results separately for different experimental groups or sample subsets, such as groups differing in baseline performance or age-related sample characteristics.

**Supplementary Appendix B. Included studies contributing non-WM outcomes**

Table S2. Study characteristics for analyses of transfer to non-WM outcomes

| **Study** | **id** | **Training task** | **Test task** | **cognitive domain** | **direction** | **g** |
| --- | --- | --- | --- | --- | --- | --- |
| Ang et al. (2015) | 1 | Cogmed | Numerical Operations Task | other | span_to_other | -0.288 |
| Ang et al. (2015) | 2 | Cogmed | Additional Fluency Task | other | span_to_other | 0.000 |
| Ang et al. (2015) | 3 | Cogmed | Subtraction Fluency Task | other | span_to_other | -0.147 |
| Ang et al. (2015) | 4 | running span, keep track | Numerical Operations Task | other | updating_to_other | 0.055 |
| Ang et al. (2015) | 5 | running span, keep track | Additional Fluency Task | other | updating_to_other | 0.250 |
| Ang et al. (2015) | 6 | running span, keep track | Subtraction Fluency Task | other | updating_to_other | 0.491 |
| Anguera et al. (2012) | 7 | dual n-back | Visuomotor Adaptation | other | updating_to_other | -0.473 |
| Booth et al. (2023) | 8 | Distributed single n-back | Relational reasoning | intelligence | updating_to_other | -0.268 |
| Booth et al. (2023) | 9 | Intensive single n-back | Relational reasoning | intelligence | updating_to_other | -0.664 |
| Bürki et al. (2014) | 10 | verbal n-back | Raven’s Progressive Matrices | intelligence | updating_to_other | 0.013 |
| Bürki et al. (2014) | 11 | verbal n-back | Raven’s Progressive Matrices | intelligence | updating_to_other | 0.123 |
| Bürki et al. (2014) | 12 | verbal n-back | Stroop Interference | other | updating_to_other | 0.171 |
| Bürki et al. (2014) | 13 | verbal n-back | Stroop Interference | other | updating_to_other | 0.157 |
| Bürki et al. (2014) | 14 | verbal n-back | Letter Comparison | other | updating_to_other | 0.160 |
| Bürki et al. (2014) | 15 | verbal n-back | Letter Comparison | other | updating_to_other | -0.259 |
| Bürki et al. (2014) | 16 | verbal n-back | Pattern Comparison | other | updating_to_other | -0.086 |
| Bürki et al. (2014) | 17 | verbal n-back | Pattern Comparison | other | updating_to_other | -0.087 |
| Bürki et al. (2014) | 18 | verbal n-back | Simple Reaction Time | other | updating_to_other | 0.100 |
| Bürki et al. (2014) | 19 | verbal n-back | Simple Reaction Time | other | updating_to_other | -0.480 |
| Cai et al. (2022) | 20 | color delay estimation | Raven’s Advanced Progressive Matrices | intelligence | span_to_other | -0.296 |
| Chang (2019) | 21 | single n-back | Geometric academic achievements | other | updating_to_other | 0.512 |
| Chang (2019) | 22 | single n-back | Geometric academic achievements | other | updating_to_other | 1.111 |
| Chen (2021) | 23 | running memory | Raven’s Standard Progressive Matrices | intelligence | updating_to_other | -0.098 |
| Chooi & Thompson (2012) | 24 | dual n-back task | Mill-Hill | other | updating_to_other | 0.661 |
| Chooi & Thompson (2012) | 25 | dual n-back task | Word Beginning and Ending | other | updating_to_other | 0.026 |
| Chooi & Thompson (2012) | 26 | dual n-back task | Colorado Perceptual Speed Test | other | updating_to_other | 0.378 |
| Chooi & Thompson (2012) | 27 | dual n-back task | Identical Picture | other | updating_to_other | 0.022 |
| Chooi & Thompson (2012) | 28 | dual n-back task | Finding A’s | other | updating_to_other | -0.360 |
| Chooi & Thompson (2012) | 29 | dual n-back task | Paper Folding | intelligence | updating_to_other | 0.684 |
| Chooi & Thompson (2012) | 30 | dual n-back task | Card Rotation | other | updating_to_other | 0.229 |
| Chooi & Thompson (2012) | 31 | dual n-back task | Mental Rotation | other | updating_to_other | -0.311 |
| Chooi & Thompson (2012) | 32 | dual n-back task | Raven's Advanced Progressive Matrices | intelligence | updating_to_other | 0.103 |
| Chooi & Thompson (2012) | 33 | dual n-back task | Mill-Hill | intelligence | updating_to_other | -0.152 |
| Chooi & Thompson (2012) | 34 | dual n-back task | Word Beginning and Ending | other | updating_to_other | -0.026 |
| Chooi & Thompson (2012) | 35 | dual n-back task | Colorado Perceptual Speed Test | other | updating_to_other | -1.347 |
| Chooi & Thompson (2012) | 36 | dual n-back task | Identical Picture | other | updating_to_other | 0.619 |
| Chooi & Thompson (2012) | 37 | dual n-back task | Finding A’s | other | updating_to_other | -0.083 |
| Chooi & Thompson (2012) | 38 | dual n-back task | Paper Folding | other | updating_to_other | -0.263 |
| Chooi & Thompson (2012) | 39 | dual n-back task | Card Rotation | other | updating_to_other | -0.317 |
| Chooi & Thompson (2012) | 40 | dual n-back task | Mental Rotation | other | updating_to_other | 0.116 |
| Chooi & Thompson (2012) | 41 | dual n-back task | Raven's Advanced Progressive Matrices | intelligence | updating_to_other | 0.262 |
| Colom et al. (2013) | 42 | visual n-back, verbal n-back, dual n-back | Raven Advanced Progressive Matrices Test (RAPM) | intelligence | updating_to_other | 0.305 |
| Colom et al. (2013) | 43 | visual n-back, verbal n-back, dual n-back | abstract reasoning subtest from the Differential Aptitude Test (DAT-AR) | intelligence | updating_to_other | 0.226 |
| Colom et al. (2013) | 44 | visual n-back, verbal n-back, dual n-back | Primary Mental Abilities Battery (PMA-R) | intelligence | updating_to_other | 0.051 |
| Colom et al. (2013) | 45 | visual n-back, verbal n-back, dual n-back | verbal reasoning subtest from the DAT (DAT-VR) | intelligence | updating_to_other | -0.306 |
| Colom et al. (2013) | 46 | visual n-back, verbal n-back, dual n-back | numerical reasoning subtest from the DAT (DAT-NR) | intelligence | updating_to_other | 0.317 |
| Colom et al. (2013) | 47 | visual n-back, verbal n-back, dual n-back | vocabulary subtest from the PMA (PMA-V) | intelligence | updating_to_other | -0.191 |
| Colom et al. (2013) | 48 | visual n-back, verbal n-back, dual n-back | Verbal flanker | other | updating_to_other | 0.002 |
| Colom et al. (2013) | 49 | visual n-back, verbal n-back, dual n-back | Numerical flanker | other | updating_to_other | -0.142 |
| Colom et al. (2013) | 50 | visual n-back, verbal n-back, dual n-back | Spatial Simon | other | updating_to_other | 0.298 |
| Dahlin et al. (2008) | 51 | running memory letters, keep track | Digit symbol | other | updating_to_other | -0.043 |
| Dahlin et al. (2008) | 52 | running memory letters, keep track | Letter fluency | other | updating_to_other | -0.058 |
| Dahlin et al. (2008) | 53 | running memory letters, keep track | Category fluency | other | updating_to_other | -0.085 |
| Dahlin et al. (2008) | 54 | running memory letters, keep track | Raven’s Advanced Progressive Matrices | intelligence | updating_to_other | 0.298 |
| Dahlin et al. (2008) | 55 | running memory letters, keep track | Digit symbol | other | updating_to_other | 0.360 |
| Dahlin et al. (2008) | 56 | running memory letters, keep track | Letter fluency | other | updating_to_other | 0.380 |
| Dahlin et al. (2008) | 57 | running memory letters, keep track | Category fluency | other | updating_to_other | 0.122 |
| Dahlin et al. (2008) | 58 | running memory letters, keep track | Raven’s Advanced Progressive Matrices | intelligence | updating_to_other | 0.056 |
| Foster et al. (2017) | 59 | running memory | Matrices | intelligence | updating_to_other | 0.219 |
| Foster et al. (2017) | 60 | running memory | Paper folding | intelligence | updating_to_other | -0.048 |
| Foster et al. (2017) | 61 | running memory | Synthetic work | other | updating_to_other | -0.058 |
| Foster et al. (2017) | 62 | running memory | Matrices | intelligence | updating_to_other | -0.192 |
| Foster et al. (2017) | 63 | running memory | Paper folding | intelligence | updating_to_other | 0.104 |
| Foster et al. (2017) | 64 | running memory | Synthetic work | other | updating_to_other | 0.351 |
| Foster et al. (2017) | 65 | operation span, symmetry span | Matrices | intelligence | span_to_other | -0.110 |
| Foster et al. (2017) | 66 | operation span, symmetry span | Paper folding | intelligence | span_to_other | -0.209 |
| Foster et al. (2017) | 67 | operation span, symmetry span | Synthetic work | other | span_to_other | -0.056 |
| Foster et al. (2017) | 68 | operation span, symmetry span | Matrices | intelligence | span_to_other | -0.112 |
| Foster et al. (2017) | 69 | operation span, symmetry span | Paper folding | intelligence | span_to_other | 0.045 |
| Foster et al. (2017) | 70 | operation span, symmetry span | Synthetic work | other | span_to_other | 0.128 |
| Hadwin & Richards (2016) | 71 | Cogmed | Stroop Interference | other | span_to_other | 1.164 |
| Harrison et al. (2013) | 72 | operation-span, symmetry-span | Raven’s Advanced Progressive Matrices (RAPM) | intelligence | span_to_other | 0.830 |
| Harrison et al. (2013) | 73 | operation-span, symmetry-span | Letter Sets | intelligence | span_to_other | 0.388 |
| Harrison et al. (2013) | 74 | operation-span, symmetry-span | Number Series | intelligence | span_to_other | -0.521 |
| Harrison et al. (2013) | 75 | simple span | Raven’s Advanced Progressive Matrices (RAPM) | intelligence | span_to_other | 1.018 |
| Harrison et al. (2013) | 76 | simple span | Letter Sets | intelligence | span_to_other | -0.598 |
| Harrison et al. (2013) | 77 | simple span | Number Series | intelligence | span_to_other | -2.132 |
| Harrison et al. (2013) | 78 | operation-span, symmetry-span | SynWin | other | span_to_other | -1.093 |
| Harrison et al. (2013) | 79 | operation-span, symmetry-span | ControlTower Primary Score | other | span_to_other | 2.079 |
| Harrison et al. (2013) | 80 | operation-span, symmetry-span | ControlTower Distract Score | other | span_to_other | -1.742 |
| Harrison et al. (2013) | 81 | simple span | SynWin | other | span_to_other | -0.600 |
| Harrison et al. (2013) | 82 | simple span | ControlTower Primary Score | other | span_to_other | 2.717 |
| Harrison et al. (2013) | 83 | simple span | ControlTower Distract Score | other | span_to_other | -2.866 |
| Heinzel et al. (2014) | 84 | single n-back | Digit Symbol | other | updating_to_other | 0.903 |
| Heinzel et al. (2014) | 85 | single n-back | Verbal Fluency | other | updating_to_other | 1.272 |
| Heinzel et al. (2014) | 86 | single n-back | Raven’s Standard Progressive Matrices | intelligence | updating_to_other | 0.592 |
| Heinzel et al. (2014) | 87 | single n-back | Figural Relations subtest (LPS) | intelligence | updating_to_other | 0.285 |
| Heinzel et al. (2014) | 88 | single n-back | Digit Symbol | other | updating_to_other | 0.486 |
| Heinzel et al. (2014) | 89 | single n-back | Verbal Fluency | other | updating_to_other | 0.078 |
| Heinzel et al. (2014) | 90 | single n-back | Raven’s Standard Progressive Matrices | intelligence | updating_to_other | 0.179 |
| Heinzel et al. (2014) | 91 | single n-back | Figural Relations subtest (LPS) | intelligence | updating_to_other | 0.334 |
| Jaeggi et al. (2010) | 92 | single n-back | Raven's Advanced Progressive Matrices | intelligence | updating_to_other | 0.494 |
| Jaeggi et al. (2010) | 93 | single n-back | Bochumer Matrices Test (BOMAT) | intelligence | updating_to_other | 0.561 |
| Jaeggi et al. (2010) | 94 | dual n-back task | Raven's Advanced Progressive Matrices | intelligence | updating_to_other | 0.752 |
| Jaeggi et al. (2010) | 95 | dual n-back task | Bochumer Matrices Test (BOMAT) | intelligence | updating_to_other | 0.292 |
| Jakoby et al. (2019) | 96 | single n-back | WAIS-Block Design test | intelligence | updating_to_other | 0.099 |
| Jakoby et al. (2019) | 97 | single n-back | RAVEN | intelligence | updating_to_other | -0.101 |
| Kang (2023) | 98 | word simple span, word complex span, sentence complex span | Addition | other | span_to_other | -0.202 |
| Kang (2023) | 99 | location simple span, location complex span | Addition | other | span_to_other | 0.145 |
| Kang (2023) | 100 | word simple span, word complex span, sentence complex span | Subtraction | other | span_to_other | 0.490 |
| Kang (2023) | 101 | location simple span, location complex span | Subtraction | other | span_to_other | 0.655 |
| Kang (2023) | 102 | word simple span, word complex span, sentence complex span | Arithmetic Completion | other | span_to_other | 0.270 |
| Kang (2023) | 103 | location simple span, location complex span | Arithmetic Completion | other | span_to_other | 0.457 |
| Kang (2023) | 104 | word simple span, word complex span, sentence complex span | Magnitude Comparison | other | span_to_other | 0.286 |
| Kang (2023) | 105 | location simple span, location complex span | Magnitude Comparison | other | span_to_other | 0.663 |
| Kang (2023) | 106 | word simple span, word complex span, sentence complex span | Number Continuation | other | span_to_other | 0.461 |
| Kang (2023) | 107 | location simple span, location complex span | Number Continuation | other | span_to_other | 0.447 |
| Kang (2023) | 108 | word simple span, word complex span, sentence complex span | Length Estimation | other | span_to_other | 0.228 |
| Kang (2023) | 109 | location simple span, location complex span | Length Estimation | other | span_to_other | 0.533 |
| Kang (2023) | 110 | word simple span, word complex span, sentence complex span | Figure Counting | other | span_to_other | -0.066 |
| Kang (2023) | 111 | location simple span, location complex span | Figure Counting | other | span_to_other | -0.168 |
| Kang (2023) | 112 | word simple span, word complex span, sentence complex span | Block Counting | other | span_to_other | 0.269 |
| Kang (2023) | 113 | location simple span, location complex span | Block Counting | other | span_to_other | 0.155 |
| Kang (2023) | 114 | word simple span, word complex span, sentence complex span | Number Connection | other | span_to_other | -0.238 |
| Kang (2023) | 115 | location simple span, location complex span | Number Connection | other | span_to_other | -0.667 |
| Kang (2023) | 116 | word simple span, word complex span, sentence complex span | Addition | other | span_to_other | 0.031 |
| Kang (2023) | 117 | location simple span, location complex span | Addition | other | span_to_other | 0.066 |
| Kang (2023) | 118 | word simple span, word complex span, sentence complex span | Subtraction | other | span_to_other | -0.027 |
| Kang (2023) | 119 | location simple span, location complex span | Subtraction | other | span_to_other | -0.311 |
| Kang (2023) | 120 | word simple span, word complex span, sentence complex span | Arithmetic Completion | other | span_to_other | -0.182 |
| Kang (2023) | 121 | location simple span, location complex span | Arithmetic Completion | other | span_to_other | 0.011 |
| Kang (2023) | 122 | word simple span, word complex span, sentence complex span | Multiplication | other | span_to_other | 0.040 |
| Kang (2023) | 123 | location simple span, location complex span | Multiplication | other | span_to_other | 0.016 |
| Kang (2023) | 124 | word simple span, word complex span, sentence complex span | Division | other | span_to_other | -0.003 |
| Kang (2023) | 125 | location simple span, location complex span | Division | other | span_to_other | 0.070 |
| Kang (2023) | 126 | word simple span, word complex span, sentence complex span | Magnitude Comparison | other | span_to_other | -0.071 |
| Kang (2023) | 127 | location simple span, location complex span | Magnitude Comparison | other | span_to_other | 0.132 |
| Kang (2023) | 128 | word simple span, word complex span, sentence complex span | Number Continuation | other | span_to_other | -0.118 |
| Kang (2023) | 129 | location simple span, location complex span | Number Continuation | other | span_to_other | -0.240 |
| Kang (2023) | 130 | word simple span, word complex span, sentence complex span | Length Estimation | other | span_to_other | 0.021 |
| Kang (2023) | 131 | location simple span, location complex span | Length Estimation | other | span_to_other | -0.157 |
| Kang (2023) | 132 | word simple span, word complex span, sentence complex span | Figure Counting | other | span_to_other | -0.106 |
| Kang (2023) | 133 | location simple span, location complex span | Figure Counting | other | span_to_other | 0.073 |
| Kang (2023) | 134 | word simple span, word complex span, sentence complex span | Block Counting | other | span_to_other | -0.505 |
| Kang (2023) | 135 | location simple span, location complex span | Block Counting | other | span_to_other | -0.244 |
| Kang (2023) | 136 | word simple span, word complex span, sentence complex span | Number Connection | other | span_to_other | 0.212 |
| Kang (2023) | 137 | location simple span, location complex span | Number Connection | other | span_to_other | 0.240 |
| Lawlor-Savage & Goghari (2016) | 138 | dual n-back | Raven’s Advanced Progressive Matrices (RAPM) | intelligence | updating_to_other | -0.131 |
| Lawlor-Savage & Goghari (2016) | 139 | dual n-back | Cattell’s Culture Fair Test (CCFT) | intelligence | updating_to_other | 0.148 |
| Lee (2014) | 140 | animals complex span | Oral Reading Fluency (R-CBM) | other | span_to_other | 0.365 |
| Lee (2014) | 141 | animals complex span | Maze-CBM:silent reading fluency and basic reading comprehension | other | span_to_other | 0.580 |
| Lee (2014) | 142 | animals complex span | WJ-III Reading Fluency | other | span_to_other | -0.113 |
| Lee (2014) | 143 | animals complex span | WJ-III Passage Comprehension | other | span_to_other | 0.326 |
| Lee (2014) | 144 | animals complex span | Gates-MacGinitie Comprehension | other | span_to_other | 0.134 |
| Linares et al. (2018) | 145 | Arithmetical updating task, Number size updating task | Raven Standard Progressive Matrices | intelligence | updating_to_other | -0.028 |
| Linares et al. (2018) | 146 | Arithmetical updating task, Number size updating task | Cattell test | intelligence | updating_to_other | 0.101 |
| Lindeløv et al. (2016) | 147 | single n-back | Raven’s Advanced Progressive Matrices (RAPM) | intelligence | updating_to_other | 0.163 |
| Lindeløv et al. (2016) | 148 | single n-back | WAIS-IV Processing Speed Index (digit-symbol coding +symbol search) | other | updating_to_other | -0.036 |
| Lindeløv et al. (2016) | 149 | single n-back | Stroop interference effect | other | updating_to_other | 0.194 |
| Lindeløv et al. (2016) | 150 | single n-back | Stroop RT | other | updating_to_other | -0.041 |
| Lindsay (2012) | 151 | single n-back | Rhyme Judgment | other | updating_to_other | 0.611 |
| Lindsay (2012) | 152 | single n-back | Rhyme Production | other | updating_to_other | 0.343 |
| Lindsay (2012) | 153 | single n-back | Phoneme Judgment | other | updating_to_other | 0.218 |
| Lindsay (2012) | 154 | single n-back | Phoneme Deletion – simple | other | updating_to_other | 0.975 |
| Lindsay (2012) | 155 | single n-back | Phoneme Deletion – complex | other | updating_to_other | 0.000 |
| Lindsay (2012) | 156 | single n-back | Phoneme Substitution – simple | other | updating_to_other | -0.409 |
| Lindsay (2012) | 157 | single n-back | Phoneme Substitution – complex | other | updating_to_other | -0.681 |
| Minear et al. (2016) | 158 | Complex span | Animal Task (verbal simple choice reaction time task) | other | span_to_other | 0.140 |
| Minear et al. (2016) | 159 | Complex span | Word Task (verbal simple choice reaction time task) | other | span_to_other | 0.269 |
| Minear et al. (2016) | 160 | Complex span | Dot Judgment (visual simple choice reaction time task) | other | span_to_other | -1.591 |
| Minear et al. (2016) | 161 | Complex span | Array Matching (visual simple choice reaction time task) | other | span_to_other | 0.793 |
| Minear et al. (2016) | 162 | Complex span | ANT-AA (flanker task-Alerting Attention) | other | span_to_other | -0.474 |
| Minear et al. (2016) | 163 | Complex span | ANT-OA (flanker task-Orienting Attention) | other | span_to_other | -0.812 |
| Minear et al. (2016) | 164 | Complex span | ANT-EA (flanker task-Executive Attention) | other | span_to_other | 0.026 |
| Minear et al. (2016) | 165 | Complex span | Simon High Conflict | other | span_to_other | -0.013 |
| Minear et al. (2016) | 166 | Complex span | Simon Low Conflict | other | span_to_other | -0.050 |
| Minear et al. (2016) | 167 | Complex span | Inferences | intelligence | span_to_other | 0.172 |
| Minear et al. (2016) | 168 | Complex span | Nonsense Syllogisms | intelligence | span_to_other | 0.305 |
| Minear et al. (2016) | 169 | Complex span | Ravens Progressive Matrices | intelligence | span_to_other | -0.349 |
| Minear et al. (2016) | 170 | Complex span | Cattell Culture Fair | intelligence | span_to_other | -0.302 |
| Minear et al. (2016) | 171 | Complex span | Mathematics Aptitude | other | span_to_other | 0.058 |
| Minear et al. (2016) | 172 | Complex span | Arithmetic Aptitude | other | span_to_other | 0.348 |
| Minear et al. (2016) | 173 | spatial n-back | Animal Task (verbal simple choice reaction time task) | other | updating_to_other | 0.098 |
| Minear et al. (2016) | 174 | spatial n-back | Word Task (verbal simple choice reaction time task) | other | updating_to_other | 0.084 |
| Minear et al. (2016) | 175 | spatial n-back | Dot Judgment (visual simple choice reaction time task) | other | updating_to_other | -1.395 |
| Minear et al. (2016) | 176 | spatial n-back | Array Matching (visual simple choice reaction time task) | other | updating_to_other | 0.930 |
| Minear et al. (2016) | 177 | spatial n-back | ANT-AA (flanker task-Alerting Attention) | other | updating_to_other | -0.279 |
| Minear et al. (2016) | 178 | spatial n-back | ANT-OA (flanker task-Orienting Attention) | other | updating_to_other | -0.960 |
| Minear et al. (2016) | 179 | spatial n-back | ANT-EA (flanker task-Executive Attention) | other | updating_to_other | 0.362 |
| Minear et al. (2016) | 180 | spatial n-back | Simon High Conflict | other | updating_to_other | 0.202 |
| Minear et al. (2016) | 181 | spatial n-back | Simon Low Conflict | other | updating_to_other | -0.089 |
| Minear et al. (2016) | 182 | spatial n-back | Inferences | intelligence | updating_to_other | -0.048 |
| Minear et al. (2016) | 183 | spatial n-back | Nonsense Syllogisms | intelligence | updating_to_other | 0.054 |
| Minear et al. (2016) | 184 | spatial n-back | Ravens Progressive Matrices | intelligence | updating_to_other | -0.512 |
| Minear et al. (2016) | 185 | spatial n-back | Cattell Culture Fair | intelligence | updating_to_other | 0.063 |
| Minear et al. (2016) | 186 | spatial n-back | Mathematics Aptitude | other | updating_to_other | 0.087 |
| Minear et al. (2016) | 187 | spatial n-back | Arithmetic Aptitude | other | updating_to_other | 0.000 |
| Oelhafen et al. (2013) | 188 | dual n-back task | ANT (Attention Network Test) – Congruent RT | other | updating_to_other | 0.476 |
| Oelhafen et al. (2013) | 189 | dual n-back task | ANT (Attention Network Test) – Incongruent RT | other | updating_to_other | 0.425 |
| Oelhafen et al. (2013) | 190 | dual n-back task | BOMAT (Bochumer Matrizentest-advanced) | intelligence | updating_to_other | -0.045 |
| Pahor et al. (2022) | 191 | n-back | Matrix Reasoning (RCT1) | intelligence | updating_to_other | 0.316 |
| Pahor et al. (2022) | 192 | n-back | Countermanding (RCT1) | other | updating_to_other | 0.110 |
| Pahor et al. (2022) | 193 | n-back | Matrix Reasoning / UCMRT (RCT2) | intelligence | updating_to_other | 0.115 |
| Pahor et al. (2022) | 194 | n-back | Countermanding (RCT2) | intelligence | updating_to_other | 0.287 |
| Pahor et al. (2022) | 195 | n-back | Matrix Reasoning / UCMRT (RCT3) | intelligence | updating_to_other | 0.105 |
| Pahor et al. (2022) | 196 | n-back | Countermanding (RCT3) | other | updating_to_other | 0.338 |
| Pahor et al. (2022) | 197 | n-back | UCancellation (RCT3) | other | updating_to_other | 0.230 |
| Pahor et al. (2022) | 198 | n-back | ETS Surface Development (RCT3) | intelligence | updating_to_other | -0.082 |
| Pahor et al. (2022) | 199 | n-back | ETS Space Relations (RCT3) | intelligence | updating_to_other | -0.257 |
| Pahor et al. (2022) | 200 | n-back | DAT Form Board (RCT3) | intelligence | updating_to_other | 0.098 |
| Pan et al. (2018) | 201 | dual n-back | Digital Shifting | other | updating_to_other | 0.378 |
| Pan et al. (2018) | 202 | dual n-back | Stroop Task | other | updating_to_other | 0.555 |
| Pergher et al. (2018) | 203 | single n-back | TOVA task (Test of Variables of Attention) | other | updating_to_other | 0.318 |
| Pergher et al. (2018) | 204 | single n-back | RAVEN (Raven’s Progressive Matrices) | intelligence | updating_to_other | 0.264 |
| Pergher et al. (2018) | 205 | single n-back | TOVA task (Test of Variables of Attention) | other | updating_to_other | 13.790 |
| Pergher et al. (2018) | 206 | single n-back | RAVEN (Raven’s Progressive Matrices) | intelligence | updating_to_other | 0.945 |
| Pugin et al. (2014) | 207 | single n-back | MAT (Matrix Reasoning task /Test of Non-verbal Intelligence) | intelligence | updating_to_other | 0.370 |
| Pugin et al. (2014) | 208 | single n-back | Stroop Task-RT | other | updating_to_other | -0.038 |
| Pugin et al. (2014) | 209 | single n-back | Flanker Task-RT | other | updating_to_other | -0.032 |
| Pugin et al. (2014) | 210 | single n-back | SST (Symbol Search Task) | other | updating_to_other | -0.085 |
| Pugin et al. (2014) | 211 | single n-back | DSS (Digit-Symbol Substitution Task) | other | updating_to_other | -0.108 |
| Redick et al. (2013) | 212 | dual n-back task | Raven’s Advanced Progressive Matrices (RAPM) | intelligence | updating_to_other | -0.179 |
| Redick et al. (2013) | 213 | dual n-back task | Raven’s Standard Progressive Matrices (RSPM) | intelligence | updating_to_other | 0.000 |
| Redick et al. (2013) | 214 | dual n-back task | Cattell Culture-Fair Test | intelligence | updating_to_other | -0.430 |
| Redick et al. (2013) | 215 | dual n-back task | Paper Folding | intelligence | updating_to_other | 0.298 |
| Redick et al. (2013) | 216 | dual n-back task | Letter Sets | intelligence | updating_to_other | 0.424 |
| Redick et al. (2013) | 217 | dual n-back task | Number Series | intelligence | updating_to_other | -0.118 |
| Redick et al. (2013) | 218 | dual n-back task | Inferences | intelligence | updating_to_other | 0.316 |
| Redick et al. (2013) | 219 | dual n-back task | Analogies | intelligence | updating_to_other | -0.132 |
| Redick et al. (2013) | 220 | dual n-back task | SynWin | other | updating_to_other | -0.387 |
| Redick et al. (2013) | 221 | dual n-back task | Control Tower | other | updating_to_other | -0.243 |
| Redick et al. (2013) | 222 | dual n-back task | ATClab (modified from Air Traffic Control lab task) | other | updating_to_other | -0.073 |
| Redick et al. (2013) | 223 | dual n-back task | Vocabulary | intelligence | updating_to_other | 0.245 |
| Redick et al. (2013) | 224 | dual n-back task | General Knowledge | intelligence | updating_to_other | -0.078 |
| Redick et al. (2013) | 225 | dual n-back task | Letter Comparison | other | updating_to_other | 0.404 |
| Redick et al. (2013) | 226 | dual n-back task | Number Comparison | other | updating_to_other | -0.104 |
| Redick et al. (2020) | 227 | operation letter span | Letter Fluency | other | span_to_other | 0.255 |
| Redick et al. (2020) | 228 | operation letter span | Category Fluency | other | span_to_other | 0.295 |
| Redick et al. (2020) | 229 | operation letter span | Nelson Denny (Nelson-Denny Reading Comprehension) | other | span_to_other | 0.185 |
| Ripp et al. (2022) | 230 | single n-back | short-APM (short version of Raven’s Advanced Progressive Matrices) | intelligence | updating_to_other | 0.305 |
| Ripp et al. (2022) | 231 | single n-back | IGT (Iowa Gambling Task) | other | updating_to_other | 0.120 |
| Schwarb et al. (2016) | 232 | single n-back | RAPM (Raven’s Advanced Progressive Matrices) | intelligence | updating_to_other | -0.124 |
| Schwarb et al. (2016) | 233 | single n-back | RAPM (Raven’s Advanced Progressive Matrices) | intelligence | updating_to_other | 0.498 |
| Schwarb et al. (2016) | 234 | single n-back | Cattell’s Culture Fair | intelligence | updating_to_other | -0.033 |
| Schwarb et al. (2016) | 235 | single n-back | Antisaccade task | other | updating_to_other | -0.336 |
| Schwarb et al. (2016) | 236 | single n-back | Flanker task | other | updating_to_other | -0.725 |
| Schwarb et al. (2016) | 237 | single n-back | RAPM (Raven’s Advanced Progressive Matrices) | intelligence | updating_to_other | 0.069 |
| Schwarb et al. (2016) | 238 | single n-back | Cattell’s Culture Fair | intelligence | updating_to_other | 0.202 |
| Schwarb et al. (2016) | 239 | single n-back | Antisaccade task | other | updating_to_other | -0.461 |
| Schwarb et al. (2016) | 240 | single n-back | Flanker task | other | updating_to_other | -0.399 |
| Schweizer et al. (2011) | 241 | dual n-back task | Raven's Progressive Matrices | intelligence | updating_to_other | 1.709 |
| Stepankova et al. (2014) | 242 | verbal n-back | Block Design (WAIS-III subset) | intelligence | updating_to_other | 0.117 |
| Stepankova et al. (2014) | 243 | verbal n-back | Matrix Reasoning (WAIS-III subset) | intelligence | updating_to_other | 0.174 |
| Stepankova et al. (2014) | 244 | verbal n-back | Block Design (WAIS-III subset) | intelligence | updating_to_other | 0.417 |
| Stepankova et al. (2014) | 245 | verbal n-back | Matrix Reasoning (WAIS-III subset) | intelligence | updating_to_other | 0.483 |
| Studer-Luethi & Meier (2021) | 246 | dual n-back | DSST (Digit Symbol Substitution Test) | other | updating_to_other | -0.011 |
| Studer-Luethi & Meier (2021) | 247 | dual n-back | Choice Reaction accuracy (acc) | other | updating_to_other | -0.381 |
| Studer-Luethi & Meier (2021) | 248 | dual n-back | Choice Reaction reaction time (rt) | other | updating_to_other | -0.060 |
| Studer-Luethi & Meier (2021) | 249 | dual n-back | Task Switching accuracy cost (acc) | other | updating_to_other | 0.069 |
| Studer-Luethi & Meier (2021) | 250 | dual n-back | Task Switching reaction time cost (rt) | other | updating_to_other | 0.062 |
| Studer-Luethi & Meier (2021) | 251 | dual n-back | RPM (Raven’s Standard Progressive Matrices) | intelligence | updating_to_other | 0.184 |
| Thompson et al. (2013) | 252 | dual n-back task | Raven’s Advanced Progressive Matrices (RAPM) | intelligence | updating_to_other | 0.095 |
| Thompson et al. (2013) | 253 | dual n-back task | Vocabulary (WASI/WAIS Vocabulary) | intelligence | updating_to_other | 0.000 |
| Thompson et al. (2013) | 254 | dual n-back task | Blocks (WASI/WAIS Block Design) | intelligence | updating_to_other | -0.221 |
| Thompson et al. (2013) | 255 | dual n-back task | Similarities (WASI/WAIS Similarities) | intelligence | updating_to_other | 0.208 |
| Thompson et al. (2013) | 256 | dual n-back task | Matrix Reasoning (WASI/WAIS Matrix Reasoning) | intelligence | updating_to_other | -0.804 |
| Thompson et al. (2013) | 257 | dual n-back task | Nelson-Denny Comprehension | other | updating_to_other | 0.000 |
| Thompson et al. (2013) | 258 | dual n-back task | Nelson-Denny Reading Rate | other | updating_to_other | -0.044 |
| Thompson et al. (2013) | 259 | dual n-back task | Digit Symbol Coding | other | updating_to_other | 0.181 |
| Thompson et al. (2013) | 260 | dual n-back task | Visual Matching (Woodcock-Johnson III Visual Matching) | other | updating_to_other | 0.000 |
| Thompson et al. (2013) | 261 | dual n-back task | Pair Cancellation (Woodcock-Johnson III Pair Cancellation) | other | updating_to_other | 0.156 |
| Von Bastian & Eschen (2016) | 262 | three complex span tasks | RAPM (Raven’s Advanced Progressive Matrices) | intelligence | span_to_other | 0.022 |
| Von Bastian & Eschen (2016) | 263 | three complex span tasks | Letter Sets Test | intelligence | span_to_other | 0.174 |
| Von Bastian & Eschen (2016) | 264 | three complex span tasks | Locations Test | intelligence | span_to_other | -0.306 |
| Von Bastian & Eschen (2016) | 265 | three complex span tasks | Diagramming Relationships | intelligence | span_to_other | -0.299 |
| Von Bastian & Eschen (2016) | 266 | three complex span tasks | Nonsense Syllogisms Test | intelligence | span_to_other | 0.043 |
| Von Bastian & Oberauer (2013) | 267 | complex span | Task switching | other | span_to_other | 0.152 |
| Von Bastian & Oberauer (2013) | 268 | complex span | Stroop task | other | span_to_other | -0.305 |
| Von Bastian & Oberauer (2013) | 269 | complex span | Flanker task | other | span_to_other | -0.300 |
| Von Bastian & Oberauer (2013) | 270 | complex span | Syllogisms | intelligence | span_to_other | -0.082 |
| Von Bastian & Oberauer (2013) | 271 | complex span | BIS-4S reasoning scale (Berlin Intelligence Structure test, short form) | intelligence | span_to_other | 0.247 |
| Wang (2019) | 272 | dual n-back | Raven's Standard Progressive Matrices (RSPM) | intelligence | updating_to_other | 0.387 |
| Wang (2019) | 273 | dual n-back | Raven's Advanced Progressive Matrices (RAPM) | intelligence | updating_to_other | 0.335 |
| Waris et al. (2015) | 274 | Selective Updating training, figure updating, dual n-back | Cattell Culture Fair Intelligence TesT (CFIT) | intelligence | updating_to_other | -0.223 |
| Waris et al. (2015) | 275 | Selective Updating training, figure updating, dual n-back | Set Shifting Composite | other | updating_to_other | 1.242 |
| Xiao (2019) | 276 | dual n-back | Raven’s Progressive Matrices | intelligence | updating_to_other | 0.570 |
| Zając-Lamparska (2024) | 277 | single n-back | Raven’s Standard Progressive Matrices in Polish standardization (TMS) | intelligence | updating_to_other | -0.177 |
| Zając-Lamparska (2024) | 278 | single n-back | Raven’s Standard Progressive Matrices in Polish standardization (TMS) | intelligence | updating_to_other | 0.297 |
| Zhong (2011) | 279 | single n-back | Raven’s Standard Progressive Matrices | intelligence | updating_to_other | -0.282 |
| Zhong (2011) | 280 | dual n-back | Raven’s Standard Progressive Matrices | intelligence | updating_to_other | 0.233 |
| Zhong (2011) | 281 | single n-back | Raven’s Standard Progressive Matrices | intelligence | updating_to_other | 0.089 |
| Zhong (2011) | 282 | dual n-back | Raven’s Standard Progressive Matrices | intelligence | updating_to_other | 0.224 |
| Zhong (2011) | 283 | single n-back | Raven’s Standard Progressive Matrices | intelligence | updating_to_other | 0.604 |
| Zhong (2011) | 284 | dual n-back | Raven’s Standard Progressive Matrices | intelligence | updating_to_other | 1.235 |
| Zhong (2011) | 285 | single n-back | Raven’s Standard Progressive Matrices | intelligence | updating_to_other | 0.755 |
| Zhong (2011) | 286 | dual n-back | Raven’s Standard Progressive Matrices | intelligence | updating_to_other | 0.365 |
| Zhong (2011) | 287 | single n-back | Chinese Language Achievement | other | updating_to_other | -0.095 |
| Zhong (2011) | 288 | dual n-back | Chinese Language Achievement | other | updating_to_other | 0.135 |
| Zhong (2011) | 289 | single n-back | Chinese Language Achievement | other | updating_to_other | 0.263 |
| Zhong (2011) | 290 | dual n-back | Chinese Language Achievement | other | updating_to_other | 0.220 |
| Zhong (2011) | 291 | single n-back | Chinese Language Achievement | other | updating_to_other | -0.104 |
| Zhong (2011) | 292 | dual n-back | Chinese Language Achievement | other | updating_to_other | 0.046 |
| Zhong (2011) | 293 | single n-back | Chinese Language Achievement | other | updating_to_other | -0.540 |
| Zhong (2011) | 294 | dual n-back | Chinese Language Achievement | other | updating_to_other | -0.385 |
| Zhong (2011) | 295 | single n-back | Mathematics Achievement | other | updating_to_other | 1.975 |
| Zhong (2011) | 296 | dual n-back | Mathematics Achievement | other | updating_to_other | 0.981 |
| Zhong (2011) | 297 | single n-back | Mathematics Achievement | other | updating_to_other | -0.171 |
| Zhong (2011) | 298 | dual n-back | Mathematics Achievement | other | updating_to_other | 0.479 |
| Zhong (2011) | 299 | single n-back | Mathematics Achievement | other | updating_to_other | 0.199 |
| Zhong (2011) | 300 | dual n-back | Mathematics Achievement | other | updating_to_other | -0.180 |
| Zhong (2011) | 301 | single n-back | Mathematics Achievement | other | updating_to_other | -0.223 |
| Zhong (2011) | 302 | dual n-back | Mathematics Achievement | other | updating_to_other | 0.980 |

Note: Each row represents one effect size included in the non-WM transfer analysis. In some studies, multiple effect sizes were extracted for the same training-task and transfer-task combination because the original article reported results separately for different experimental groups or sample subsets, such as groups differing in baseline performance or age-related sample characteristics.

# Supplementary Appendix C. Subtype analyses of updating and span tasks

| Table S3. Primary moderator analysis by updating and span subtypes | | | | | | | | |
| --- | --- | --- | --- | --- | --- | --- | --- | --- |
| Moderator | *m* | *k* | *g* | *SE* | *p* | 95% CI | *F* | *p* |
| single update group | 35 | 147 |  |  |  |  | 2.213 | .139 |
| single updating to span | 24 | 104 | 0.169^***^ | .049 | <.001 | [.073, .265] |  |  |
| span to single updating | 16 | 43 | 0.053 | .066 | .422 | [-.077, .184] |  |  |
| dual update group | - | - |  |  |  |  | - | - |
| dual updating to span | 18 | 50 | 0.189^***^ | .052 | <.001 | [.086, .292] |  |  |
| span to dual updating | - | - | - | - | - | - |  |  |
|  |  |  |  |  |  |  |  |  |
| complex span group | 30 | 75 |  |  |  |  | 0.726 | .397 |
| updating to complex span | 26 | 61 | 0.121^*^ | .049 | .017 | [.022, .219] |  |  |
| complex span to updating | 7 | 14 | 0.035 | .092 | .707 | [-.149, .218] |  |  |
| simple span group | 37 | 113 |  |  |  |  | 1.622 | .205 |
| updating to simple span | 30 | 100 | 0.229^***^ | .049 | <.001 | [.133, .326] |  |  |
| simple span to updating | 8 | 13 | 0.079 | .108 | .469 | [-.136, .294] |  |  |

Note: *m* = number of studies; *k* = number of effect sizes; *g* = estimated effect size. For span-training studies, all eligible updating transfer outcomes involved single updating tasks. No eligible studies examined transfer from span training to dual updating tasks; therefore, the corresponding row is marked with “-”.

^+^: *p* < .10; ^*^: *p* < .05; ^**^: *p* < .01; ^***^: *p* < .001

# Supplementary Appendix D. Restricted analysis of intelligence-related outcomes

| Table S4. Moderator analysis of transfer to intelligence-related outcomes | | | | | | | | |
| --- | --- | --- | --- | --- | --- | --- | --- | --- |
| Moderator | *m* | *k* | *g* | *SE* | *p* | 95% CI | *F* | *p* |
| Direction | 34 | 116 |  |  |  |  | 4.936 | .028^*^ |
| updating to IQ | 30 | 94 | 0.117^***^ | .034 | <.001 | [.050, .184] |  |  |
| span to IQ | 6 | 22 | -0.054 | .069 | .436 | [-.191, .083] |  |  |

Note: *m* = number of studies; *k* = number of effect sizes; *g* = estimated effect size;

^+^: *p* < .10; ^*^: *p* < .05; ^**^: *p* < .01; ^***^: *p* < .001

# Supplementary Appendix E. Alternative training-dose specifications

| Table S5. Secondary moderator analyses under alternative training-dose thresholds | | | | | | | | | |
| --- | --- | --- | --- | --- | --- | --- | --- | --- | --- |
| Analysis type | Moderator | *m* | *k* | *g* | *SE* | *p* | 95% CI | *F* | *p* |
| Training dosage | |  |  |  |  |  |  |  |  |
| low-dosage  (hour<=5 h) | direction | 20 | 59 |  |  |  |  | 4.551 | .037^*^ |
|  | updating to span | 14 | 35 | 0.200^**^ | .071 | .007 | [.057, .342] |  |  |
|  | span to updating | 6 | 24 | -0.034 | .083 | .686 | [-.200, .133] |  |  |
| high-dosage  (hour>5 h) | direction | 38 | 145 |  |  |  |  | 0.490 | .485 |
|  | updating to span | 32 | 126 | 0.178^***^ | .042 | <.001 | [.094, .262] |  |  |
|  | span to updating | 10 | 19 | 0.113 | .087 | .196 | [-.059, .285] |  |  |
|  |  |  |  |  |  |  |  |  |  |
| Training dosage | |  |  |  |  |  |  |  |  |
| low-dosage  (hour<=7 h) | direction | 31 | 112 |  |  |  |  | 2.353 | .128 |
|  | updating to span | 25 | 84 | 0.166^***^ | .050 | .001 | [.067, .265] |  |  |
|  | span to updating | 7 | 28 | 0.013 | .088 | .883 | [-.162, .188] |  |  |
| high-dosage  (hour>7 h) | direction | 29 | 92 |  |  |  |  | 2.274 | .135 |
|  | updating to span | 22 | 77 | 0.228^***^ | .053 | <.001 | [.123, .333] |  |  |
|  | span to updating | 9 | 15 | 0.065 | .096 | .501 | [-.126, .255] |  |  |
|  | |  |  |  |  |  |  |  |  |
| Training dosage | |  |  |  |  |  |  |  |  |
| low-dosage  (hour<=10 h) | direction | 42 | 163 |  |  |  |  | 1.746 | .188 |
|  | updating to span | 33 | 126 | 0.163^***^ | .045 | <.001 | [.075, .251] |  |  |
|  | span to updating | 12 | 37 | 0.054 | .074 | .463 | [-.091, .200] |  |  |
| high-dosage  (hour>10 h) | direction | 13 | 41 |  |  |  |  | 1.478 | .231 |
|  | updating to span | 10 | 35 | 0.220^**^ | .063 | .001 | [.094, .347] |  |  |
|  | span to updating | 4 | 6 | 0.043 | .132 | .744 | [-.223, .309] |  |  |

Note: *m* = number of studies; *k* = number of effect sizes; *g* = estimated effect size;

^+^: *p* < .10; ^*^: *p* < .05; ^**^: *p* < .01; ^***^: *p* < .001

| Table S6. Secondary moderator analyses under different training dosage conditions | | | | | | | | | |
| --- | --- | --- | --- | --- | --- | --- | --- | --- | --- |
| Analysis type | Moderator | *m* | *k* | *g* | *SE* | *p* | 95% CI | *F* | *p* |
| Training dosage | |  |  |  |  |  |  |  |  |
| hour<=5 h | direction | 20 | 59 |  |  |  |  | 4.551 | .037^*^ |
|  | updating to span | 14 | 35 | 0.200^**^ | .071 | .007 | [.057, .342] |  |  |
|  | span to updating | 6 | 24 | -0.034 | .083 | .686 | [-.200, .133] |  |  |
|  |  |  |  |  |  |  |  |  |  |
| 5<hour<=7 h | direction | 12 | 53 |  |  |  |  | 0.117 | .734 |
|  | updating to span | 12 | 49 | 0.140^+^ | .076 | .073 | [-.014, .293] |  |  |
|  | span to updating | 1 | 4 | 0.218 | .229 | .952 | [-.241, .677] |  |  |
|  |  |  |  |  |  |  |  |  |  |
| 7<hour<=10 h | direction | 17 | 51 |  |  |  |  | 0.979 | .327 |
|  | updating to span | 13 | 42 | 0.237^**^ | .085 | .008 | [.065, .408] |  |  |
|  | span to updating | 5 | 9 | 0.077 | .140 | .582 | [-.203, .358] |  |  |
|  |  |  |  |  |  |  |  |  |  |
| hour>10 h | direction | 13 | 41 |  |  |  |  | 1.478 | .231 |
|  | updating to span | 10 | 35 | 0.220^***^ | .063 | .001 | [.094, .347] |  |  |
|  | span to updating | 4 | 6 | 0.043 | .132 | .744 | [-.223, .309] |  |  |

Note: *m* = number of studies; *k* = number of effect sizes; *g* = estimated effect size;

^+^: *p* < .10; ^*^: *p* < .05; ^**^: *p* < .01; ^***^: *p* < .001

# Supplementary Appendix F. Extended developmental-stage analyses

| Table S7. Secondary moderator analyses by detailed developmental stage | | | | | | | | | |
| --- | --- | --- | --- | --- | --- | --- | --- | --- | --- |
| Analysis type | Moderator | *m* | *k* | *g* | *SE* | *p* | 95% CI | *F* | *p* |
| children | direction | 9 | 47 |  |  |  |  | 18.19 | <.001^***^ |
|  | updating to span | 5 | 26 | 0.362^***^ | .076 | <.001 | [.209, .516] |  |  |
|  | span to updating | 5 | 21 | -0.056 | .062 | .370 | [-.180, .068] |  |  |
| adults | direction | 42 | 131 |  |  |  |  | 0.777 | .380 |
|  | updating to span | 34 | 109 | 0.161^***^ | .042 | <.001 | [.077, .244] |  |  |
|  | span to updating | 11 | 22 | 0.086 | .078 | .272 | [-.068, .240] |  |  |
| older | direction | 7 | 21 |  |  |  |  | - | - |
|  | updating to span | 7 | 21 | 0.109 | .130 | .412 | [-.162, .380] |  |  |
|  | span to updating | - | - | - | - | - | - |  |  |

Note: *m* = number of studies; *k* = number of effect sizes; *g* = estimated effect size;

^+^: *p* < .10; ^*^: *p* < .05; ^**^: *p* < .01; ^***^: *p* < .001

# Supplementary Appendix G. Stimulus-domain congruence analyses

| Table S8. Secondary moderator analyses by stimulus-domain congruence | | | | | | | | | |
| --- | --- | --- | --- | --- | --- | --- | --- | --- | --- |
| Analysis type | Moderator | *m* | *k* | *g* | *SE* | *p* | 95% CI | *F* | *p* |
| across-domain | direction | 14 | 39 |  |  |  |  | 2.663 | .111 |
|  | updating to span | 9 | 26 | 0.165^+^ | .085 | .060 | [-.007, .338] |  |  |
|  | span to updating | 6 | 13 | -0.058 | .113 | .614 | [-.287, .172] |  |  |
| within-domain | direction | 20 | 58 |  |  |  |  | 1.804 | .185 |
|  | updating to span | 13 | 41 | 0.246^**^ | .073 | .001 | [.100, .391] |  |  |
|  | span to updating | 8 | 17 | 0.081 | .104 | .438 | [-.127, .289] |  |  |

Note: *m* = number of studies; *k* = number of effect sizes; *g* = estimated effect size;

^+^: *p* < .10; ^*^: *p* < .05; ^**^: *p* < .01; ^***^: *p* < .001

# Supplementary Appendix H. Alternative task coding analyses

| Table S9. Moderator analysis under alternative coding of storage versus manipulation | | | | | | | | |
| --- | --- | --- | --- | --- | --- | --- | --- | --- |
| Moderator | *m* | *k* | *g* | *SE* | *p* | 95% CI | *F* | *p* |
| Direction | 29 | 64 |  |  |  |  | 0.426 | 0.516 |
| manipulation to storage | 25 | 57 | 0.191^**^ | .064 | .004 | [.064, .318] |  |  |
| storage to manipulation | 4 | 7 | 0.163 | .066 | .637 | [-.248, .402] |  |  |

Note: *m* = number of studies; *k* = number of effect sizes; *g* = estimated effect size;

^+^: *p* < .10; ^*^: *p* < .05; ^**^: *p* < .01; ^***^: *p* < .001

# Supplementary Appendix I. Sensitivity Analysis

| Table S10. Primary and Secondary Moderator Analyses of within-WM transfer | | | | | | | | |  |
| --- | --- | --- | --- | --- | --- | --- | --- | --- | --- |
| Group | Moderator | *m* | *k* | *g* | *SE* | *p* | 95% CI | *F* | *p* |
| **Primary moderator analysis** | | | |  |  |  |  |  |  |
| Full model | direction | 53 | 200 |  |  |  |  | 2.844 | .093^+^ |
|  | updating to span | 41 | 157 | 0.172^***^ | .039 | <.001 | [.096, .248] |  |  |
|  | span to updating | 16 | 43 | 0.049 | .064 | .444 | [-.078,.176] |  |  |
|  |  |  |  |  |  |  |  |  |  |
| **Secondary moderator analysis** | | | | | | | | | |
| Training dosage | |  |  |  |  |  |  |  |  |
| low-dosage  (hour≤5 h) | direction | 20 | 59 |  |  |  |  | 4.551 | .037^*^ |
|  | updating to span | 14 | 35 | 0.200^**^ | .071 | .007 | [.057, .342] |  |  |
|  | span to updating | 6 | 24 | -0.034 | .083 | .686 | [-.200, .133] |  |  |
| high-dosage  (hour>5h) | direction | 37 | 141 |  |  |  |  | 0.380 | .539 |
|  | updating to span | 31 | 122 | 0.172^***^ | .045 | <.001 | [.083, .261] |  |  |
|  | span to updating | 10 | 19 | 0.114 | .088 | .196 | [-.060, .289] |  |  |
|  | |  |  |  |  |  |  |  |  |
| Developmental stage | |  |  |  |  |  |  |  |  |
| non-adults | direction | 9 | 47 |  |  |  |  | 18.19 | <.001^***^ |
|  | updating to span | 5 | 26 | 0.362^***^ | .076 | <.001 | [.209, .516] |  |  |
|  | span to updating | 5 | 21 | -0.056 | .062 | .370 | [-.180, .068] |  |  |
| adults | direction | 43 | 148 |  |  |  |  | 0.553 | .458 |
|  | updating to span | 35 | 126 | 0.151^***^ | .042 | <.001 | [.068, .234] |  |  |
|  | span to updating | 11 | 22 | 0.087 | .079 | .273 | [-.069, .243] |  |  |
|  |  |  |  |  |  |  |  |  |  |
| Training domain | |  |  |  |  |  |  |  |  |
| verbal stimuli | direction | 11 | 38 |  |  |  |  | 2.600 | .116 |
|  | updating to span | 7 | 25 | 0.267^**^ | .080 | .002 | [.105, .428] |  |  |
|  | span to updating | 4 | 13 | 0.064 | .097 | .516 | [-.133, .261] |  |  |
| nonverbal stimuli | direction | 16 | 55 |  |  |  |  | 1.446 | .235 |
|  | updating to span | 10 | 38 | 0.179 | .114 | .124 | [-.051, .409] |  |  |
|  | span to updating | 6 | 17 | -0.042 | .144 | .771 | [-.331, .246] |  |  |

Note: *m* = number of studies; *k* = number of effect sizes; *g* = estimated effect size;

^+^: *p* < .10; ^*^: *p* < .05; ^**^: *p* < .01; ^***^: *p* < .001

# References

References marked with an asterisk indicate studies included in the present meta-analyses.

*Ang, S. Y., Lee, K., Cheam, F., Poon, K., & Koh, J. (2015). Updating and working memory training: Immediate improvement, long-term maintenance, and generalisability to non-trained tasks. *Journal of Applied Research in Memory and Cognition*, *4*(2), 121–128. https://doi.org/10.1016/j.jarmac.2015.03.001

*Anguera, J. A., Bernard, J. A., Jaeggi, S. M., Buschkuehl, M., Benson, B. L., Jennett, S., Humfleet, J., Reuter-Lorenz, P. A., Jonides, J., & Seidler, R. D. (2012). The effects of working memory resource depletion and training on sensorimotor adaptation. *Behavioural Brain Research*, *228*(1), 107–115. https://doi.org/10.1016/j.bbr.2011.11.040

*Booth, S. J., Brown, L. J. E., Taylor, J. R., & Pobric, G. (2023). Experimental investigation of training schedule on home-based working memory training in healthy older adults. *Frontiers in Psychology*, *14*, 1165275. https://doi.org/10.3389/fpsyg.2023.1165275

*Bürki, C. N., Ludwig, C., Chicherio, C., & De Ribaupierre, A. (2014). Individual differences in cognitive plasticity: An investigation of training curves in younger and older adults. *Psychological Research*, *78*(6), 821–835. https://doi.org/10.1007/s00426-014-0559-3

*Cai, Y., Yang, C., Wang, S., & Xue, G. (2022). The Neural Mechanism Underlying Visual Working Memory Training and Its Limited Transfer Effect. *Journal of Cognitive Neuroscience*, *34*(11), 2082–2099. https://doi.org/10.1162/jocn_a_01897

*Chang H. (2019). *Shi jue gong zuo ji yi xun lian dui bu tong ren zhi fang shi chu zhong sheng ji he cheng ji de ying xiang* [The influence of visual working memory training on geometry scores of junior high school students with different cognitive styles] [Master’s thesis]. Shandong Normal University.

*Chen M. D. (2021). *Huodong jiyi xunlian dui gongzuo jiyi shuaxin he rongliang de yingxiang* [The effect of running memory training on working memory updating and capacity] [Master’s thesis]. Shaanxi Normal University.

*Chooi, W.-T., & Thompson, L. A. (2012). Working memory training does not improve intelligence in healthy young adults. *Intelligence*, *40*(6), 531–542. https://doi.org/10.1016/j.intell.2012.07.004

*Colom, R., Román, F. J., Abad, F. J., Shih, P. C., Privado, J., Froufe, M., Escorial, S., Martínez, K., Burgaleta, M., Quiroga, M. A., Karama, S., Haier, R. J., Thompson, P. M., & Jaeggi, S. M. (2013). Adaptive n-back training does not improve fluid intelligence at the construct level: Gains on individual tests suggest that training may enhance visuospatial processing. *Intelligence*, *41*(5), 712–727. https://doi.org/10.1016/j.intell.2013.09.002

*Dahlin, E., Nyberg, L., Bäckman, L., & Neely, A. S. (2008). Plasticity of executive functioning in young and older adults: Immediate training gains, transfer, and long-term maintenance. *Psychology and Aging*, *23*(4), 720–730. https://doi.org/10.1037/a0014296

*Fellman, D., Jylkkä, J., Waris, O., Soveri, A., Ritakallio, L., Haga, S., Salmi, J., Nyman, T. J., & Laine, M. (2020). The role of strategy use in working memory training outcomes. *Journal of Memory and Language*, *110*, 104064. https://doi.org/10.1016/j.jml.2019.104064

*Foster, J. L., Harrison, T. L., Hicks, K. L., Draheim, C., Redick, T. S., & Engle, R. W. (2017). Do the effects of working memory training depend on baseline ability level? *Journal of Experimental Psychology: Learning, Memory, and Cognition*, *43*(11), 1677–1689. https://doi.org/10.1037/xlm0000426

*Hadwin, J. A., & Richards, H. J. (2016). Working memory training and CBT reduces anxiety symptoms and attentional biases to threat: A preliminary study. *Frontiers in Psychology*, *7*. https://doi.org/10.3389/fpsyg.2016.00047

*Harrison, T. L., Shipstead, Z., Hicks, K. L., Hambrick, D. Z., Redick, T. S., & Engle, R. W. (2013). Working memory training may increase working memory capacity but not fluid intelligence. *Psychological Science*, *24*(12), 2409–2419. https://doi.org/10.1177/0956797613492984

*Heinzel, S., Schulte, S., Onken, J., Duong, Q.-L., Riemer, T. G., Heinz, A., Kathmann, N., & Rapp, M. A. (2014). Working memory training improvements and gains in non-trained cognitive tasks in young and older adults. *Aging, Neuropsychology, and Cognition*, *21*(2), 146–173. https://doi.org/10.1080/13825585.2013.790338

*Holmes, J., Woolgar, F., Hampshire, A., & Gathercole, S. E. (2019). Are working memory training effects paradigm-specific? *Frontiers in Psychology*, *10*, 1103. https://doi.org/10.3389/fpsyg.2019.01103

*Jaeggi, S. M., Buschkuehl, M., & Jonides, J. (2009). *Working memory training and transfer*. ONR contractor’s meeting, Arlington, VA.

*Jaeggi, S. M., Buschkuehl, M., Jonides, J., & Perrig, W. J. (2008). Improving fluid intelligence with training on working memory. *Proceedings of the National Academy of Sciences*, *105*(19), 6829–6833. https://doi.org/10.1073/pnas.0801268105

*Jaeggi, S. M., Studer-Luethi, B., Buschkuehl, M., Su, Y.-F., Jonides, J., & Perrig, W. J. (2010). The relationship between n-back performance and matrix reasoning—Implications for training and transfer. *Intelligence*, *38*(6), 625–635. https://doi.org/10.1016/j.intell.2010.09.001

*Jakoby, H., Raviv, O., Jaffe-Dax, S., Lieder, I., & Ahissar, M. (2019). Auditory frequency discrimination is correlated with linguistic skills, but its training does not improve them or other pitch discrimination tasks. *Journal of Experimental Psychology: General*, *148*(11), 1953–1971. https://doi.org/10.1037/xge0000573

*Kang J. (2023). *Butong gongzuo jiyi chengfen yu xiaoxuesheng shuxue nengli de guanxi: Zhuizong he xunlian yanjiu* [The relationship between different working memory domains and pupils’ mathematical abilities: A longitudinal and training study] [Master’s thesis]. Zhejiang University

*Lawlor-Savage, L., & Goghari, V. M. (2016). Dual n-back working memory training in healthy adults: A randomized comparison to processing speed training. *PLOS ONE*, *11*(4), e0151817. https://doi.org/10.1371/journal.pone.0151817

*Lee, S. E. (2014). *The impact of working memory training on third-grade students’ reading fluency and reading comprehension performance* [Doctoral thesis]. Southern Illinois University. https://opensiuc.lib.siu.edu/dissertations/958/

*Lilienthal, L., Tamez, E., Shelton, J. T., Myerson, J., & Hale, S. (2013). Dual n-back training increases the capacity of the focus of attention. *Psychonomic Bulletin & Review*, *20*(1), 135–141. https://doi.org/10.3758/s13423-012-0335-6

*Linares, R., Borella, E., Lechuga, M. T., Carretti, B., & Pelegrina, S. (2018). Training working memory updating in young adults. *Psychological Research*, *82*(3), 535–548. https://doi.org/10.1007/s00426-017-0843-0

*Linares, R., Borella, E., Lechuga, M. T., Carretti, B., & Pelegrina, S. (2019). Nearest transfer effects of working memory training: A comparison of two programs focused on working memory updating. *PLOS ONE*, *14*(2), e0211321. https://doi.org/10.1371/journal.pone.0211321

*Lindeløv, J. K., Dall, J. O., Kristensen, C. D., Aagesen, M. H., Olsen, S. A., Snuggerud, T. R., & Sikorska, A. (2016). Training and transfer effects of N-back training for brain-injured and healthy subjects. *Neuropsychological Rehabilitation*, *26*(5–6), 895–909. https://doi.org/10.1080/09602011.2016.1141692

*Lindsay, J. R. (2012). Everything i need to know i learned before elementary school: The relationships between phoneme awareness, letter knowledge, and working memory [Doctoral thesis]. University of California, Irvine.

*Minear, M., Brasher, F., Guerrero, C. B., Brasher, M., Moore, A., & Sukeena, J. (2016). A simultaneous examination of two forms of working memory training: Evidence for near transfer only. *Memory & Cognition*, *44*(7), 1014–1037. https://doi.org/10.3758/s13421-016-0616-9

*Oelhafen, S., Nikolaidis, A., Padovani, T., Blaser, D., Koenig, T., & Perrig, W. J. (2013). Increased parietal activity after training of interference control. *Neuropsychologia*, *51*(13), 2781–2790. https://doi.org/10.1016/j.neuropsychologia.2013.08.012

*Pahor, A., Seitz, A. R., & Jaeggi, S. M. (2022). Near transfer to an unrelated N-back task mediates the effect of N-back working memory training on matrix reasoning. *Nature Human Behaviour*, *6*(9), 1243–1256. https://doi.org/10.1038/s41562-022-01384-w

*Pan D., Wang D., & Li X. (2018). Jiyu shouji APP de shuangwei n-back xunlian de renzhi yu qingxu xiaoyi [Cognitive and emotional benefits of emotional dual dimension n-back training based on an APP]. *Acta Psychologica Sinica*, *50*(10), 1105–1119.

*Pergher, V., Wittevrongel, B., Tournoy, J., Schoenmakers, B., & Van Hulle, M. M. (2018). N‐back training and transfer effects revealed by behavioral responses and EEG. *Brain and Behavior*, *8*(11), e01136. https://doi.org/10.1002/brb3.1136

*Pugin, F., Metz, A. J., Stauffer, M., Wolf, M., Jenni, O. G., & Huber, R. (2014). Working memory training shows immediate and long-term effects on cognitive performance in children. *F1000Research*, *3*, 82. https://doi.org/10.12688/f1000research.3665.2

*Qi, X., Fu, J., Gu, Y., Xue, G., & Cai, Y. (2024). *The Nature of Visual Short-Term Memory Training Transfer: Evidence from A Systematical Transfer Test*. PsychArchives. https://doi.org/10.23668/psycharchives.14681

*Qi, X. Y. (2024). *Shijue gongzuo jiyi xunlian tisheng de shizhi jiqi qianyi guocheng* [The nature and process of visual spatial working memory training transfer] [Unpublished master’s thesis]. Zhejiang University.

*Rass, O., Schacht, R. L., Buckheit, K., Johnson, M. W., Strain, E. C., & Mintzer, M. Z. (2015). A randomized controlled trial of the effects of working memory training in methadone maintenance patients. *Drug and Alcohol Dependence*, *156*, 38–46. https://doi.org/10.1016/j.drugalcdep.2015.08.012

*Redick, T. S., Shipstead, Z., Harrison, T. L., Hicks, K. L., Fried, D. E., Hambrick, D. Z., Kane, M. J., & Engle, R. W. (2013). No evidence of intelligence improvement after working memory training: A randomized, placebo-controlled study. *Journal of Experimental Psychology: General*, *142*(2), 359–379. https://doi.org/10.1037/a0029082

*Redick, T. S., Wiemers, E. A., & Engle, R. W. (2020). The role of proactive interference in working memory training and transfer. *Psychological Research*, *84*(6), 1635–1654. https://doi.org/10.1007/s00426-019-01172-8

*Ripp, I., Emch, M., Wu, Q., Lizarraga, A., Udale, R., Von Bastian, C. C., Koch, K., & Yakushev, I. (2022). Adaptive working memory training does not produce transfer effects in cognition and neuroimaging. *Translational Psychiatry*, *12*(1), 512. https://doi.org/10.1038/s41398-022-02272-7

*Schwarb, H., Nail, J., & Schumacher, E. H. (2016). Working memory training improves visual short-term memory capacity. *Psychological Research*, *80*(1), 128–148. https://doi.org/10.1007/s00426-015-0648-y

*Schweizer, S., Hampshire, A., & Dalgleish, T. (2011). Extending Brain-Training to the Affective Domain: Increasing Cognitive and Affective Executive Control through Emotional Working Memory Training. *PLoS ONE*, *6*(9), e24372. https://doi.org/10.1371/journal.pone.0024372

*Song J., Zhao W., Zhang Q. M., & Li J. (2020). Daxuesheng gongzuo jiyi guangdu xunlian qianyi xiaoying de xingweixue yu yingxiangxue yanjiu [Transfer effect of working memory span training in college students:evidence from behavioral and magnetic resonance imaging]. *Chinese Journal of Behavioral Medicine and Brain Science*, *29*(10), 909–914. https://doi.org/10.3760/cma.j.cn371468-20200506-01339

*Soveri, A., Karlsson, E. P. A., Waris, O., Grönholm-Nyman, P., & Laine, M. (2017). Pattern of Near Transfer Effects Following Working Memory Training With a Dual N-Back Task. *Experimental Psychology*, *64*(4), 240–252. https://doi.org/10.1027/1618-3169/a000370

*Stepankova, H., Lukavsky, J., Buschkuehl, M., Kopecek, M., Ripova, D., & Jaeggi, S. M. (2014). The malleability of working memory and visuospatial skills: A randomized controlled study in older adults. *Developmental Psychology*, *50*(4), 1049–1059. https://doi.org/10.1037/a0034913

*Studer-Luethi, B., & Meier, B. (2021). Is Training with the N-Back Task More Effective Than with Other Tasks? N-Back vs. Dichotic Listening vs. Simple Listening. *Journal of Cognitive Enhancement*, *5*(4), 434–448. https://doi.org/10.1007/s41465-020-00202-3

*Tan, A. S. L., Lau, R. C., Anderson, P. J., Gathercole, S., Bellgrove, M. A., Wiley, J. F., & Spencer-Smith, M. M. (2024). Exploring working memory capacity and efficiency processes to understand working memory training outcomes in primary school children. *Journal of Cognition*, *7*(1), 23. https://doi.org/10.5334/joc.348

*Thompson, T. W., Waskom, M. L., Garel, K.-L. A., Cardenas-Iniguez, C., Reynolds, G. O., Winter, R., Chang, P., Pollard, K., Lala, N., Alvarez, G. A., & Gabrieli, J. D. E. (2013). Failure of Working Memory Training to Enhance Cognition or Intelligence. *PLoS ONE*, *8*(5), e63614. https://doi.org/10.1371/journal.pone.0063614

*Von Bastian, C. C., & Eschen, A. (2016). Does working memory training have to be adaptive? *Psychological Research*, *80*(2), 181–194. https://doi.org/10.1007/s00426-015-0655-z

*Von Bastian, C. C., & Oberauer, K. (2013). Distinct transfer effects of training different facets of working memory capacity. *Journal of Memory and Language*, *69*(1), 36–58. https://doi.org/10.1016/j.jml.2013.02.002

*Wang H. (2019). *Liyong jiyi gongdian he N-back yanjiu xunlian dui xingwei he danao shenjing huodong de yingxiang* [The influence of working memory training on behavior and brain neural activity using the method of loci and N-back] [Master’s thesis]. University of Electronic Science and Technology of China.

*Waris, O., Soveri, A., & Laine, M. (2015). Transfer after Working Memory Updating Training. *PLOS ONE*, *10*(9), e0138734. https://doi.org/10.1371/journal.pone.0138734

*Xiao X. (2019). *Yong N-Back renwu xunlian gongzuo jiyi de shiyan yanjiu* [An experimental study on training working memory with N-Back task] [Master’s thesis]. Yunnan Normal University.

*Zając-Lamparska, L. (2024). Limited training and transfer effects in older and young adults who participated in 12 sessions of process-based working memory training. A three-armed pretest-posttest design study. *BMC Research Notes*, *17*(1), 181. https://doi.org/10.1186/s13104-024-06844-2

*Zhong C. J. (2011). *Gongzuo jiyi yu zhuyi de xunlian dui xiaoxuesheng liuti zhili de yingxiang* [The effect of training working memory and attention on pupils’ fluid intelligence] [Master’s thesis]. Southwest University.
